# Shedding Light on Patterns of Unconventional Expression of Opsin Genes in *Hydra vulgaris*

**DOI:** 10.1093/icb/icaf100

**Published:** 2025-06-23

**Authors:** Marina I Stoilova, Natasha Picciani, Aide Macias-Muñoz, Todd H Oakley

**Affiliations:** Department of Ecology, Evolution, and Marine Biology, University of California, Santa Barbara, CA 93106, USA; Department of Ecology and Evolutionary Biology, University of Kansas, Lawrence, KS 66045, USA; Department of Ecology, Evolution, and Marine Biology, University of California, Santa Barbara, CA 93106, USA; Department of Ecology, Evolution, and Marine Biology, University of California, Santa Barbara, CA 93106, USA; Department of Ecology and Evolutionary Biology, University of California, Santa Cruz, CA 95064, USA; Department of Ecology, Evolution, and Marine Biology, University of California, Santa Barbara, CA 93106, USA

## Abstract

Opsins are G-protein-coupled receptors often expressed in neuronal photoreceptor cells and used for light detection in most animals, including cnidarians like corals, jellyfish, and anemones. Opsins may also be expressed in non-neuronal cell types, where they may confer light sensitivity. For example, opsins might be involved in pre-neural phototaxis of larval box jellyfish. However, the overall extent of non-neuronal expression of opsins is not well understood, despite the potential for identifying additional light or opsin-mediated organismal functions. To investigate the prevalence of non-neuronal opsin expression in a cnidarian, we analyzed published data from *Hydra vulgaris*, a freshwater hydroid that responds to light despite lacking distinct photosensory structures such as eyes. We quantified opsin expression across *Hydra* cell types and states of cell differentiation using published single-cell RNA sequencing (scRNA-seq) data and assay for transposase-accessible chromatin sequencing data. We identified 45 opsin transcripts in *Hydra* expressed in neuronal and non-neuronal cell types, as well as across inferred states of cell differentiation. We found a wider diversity of opsin gene transcripts in neuronal cell types, predominantly in fully differentiated cells. In contrast, we detected fewer opsin transcripts in non-neuronal cell types, and they were expressed from stem cell to progenitor cell to fully differentiated cell state—all within the same inferred cell type. These opsin transcripts appear to be expressed at higher levels in ectodermal epithelial cells near the head organizer of *Hydra* (a key developmental patterning region) and share transcription factor binding motifs with development genes such as Six, Otx, Ptx, Rfx4, and Hxa. Overall, we outline an array of opsin gene transcripts, their expression, and open chromatin patterns across cell type diversity in *Hydra*, and highlight potential co-regulatory relationships that may pave the way for future work on unconventional roles for opsin genes in *Hydra*.

## Introduction

Opsins are involved in the first step of phototransduction cascades, which convert light to an electrochemical signal that can be communicated between cells (Arshavsky et al. 2002). Based on its classic light-sensing function, we might expect opsin expression to be diagnostic for photoreceptor cells, specialized neurons for light detection. But opsins serve roles other than photoreception, including thermoreception, lipid transport, or the photoisomerization of retinal (the pigment that binds to opsin receptors) (Hao and Fong 1999; Leung and Montell 2017; Feuda et al. 2022). Our understanding of this classic opsin-photoreceptor cell association has mostly relied on approaches, such as physiological and genetic experiments and *in situ* hybridization, which are labor-intensive, require the identification of the cells by their localization and morphology, and require *a priori* knowledge of gene sequences for probe design. More recently, tissue or whole animal transcriptomics quantify gene expression without *a priori* knowledge of the specific genes, but fail to disentangle cell type heterogeneity. With single-cell technologies on the rise, we can now begin to explore more efficiently, which individual cell types express opsins and in which developmental cell states.

Extraocular photoreception is a known phenomenon across the animal tree of life (Ramirez et al. 2011), with visual phototransduction components commonly found in the central nervous system as well as non-neuronal tissues such as chromatophores and muscle cells in cephalopods or dermal photoreceptors in hogfish (Kingston et al. 2015; Schweikert et al. 2023). In the hydrozoan cnidarian *Clytia hemisphaerica*, specialized neural cells in the gonad ectoderm express opsins to mediate spawning (Quiroga Artigas et al. 2018). Among other cnidarians (sea anemones, jellyfish, and corals), cells and tissues other than neurons also respond to light, likely via opsin-mediated phototransduction cascades but this has not been directly established with functional testing in most cases. The planula larvae of the jellyfish *Tripedalia cystophora* may sense light without the presence of a nervous system or neurons (Nordström et al. 2003). In the sea anemone *Calamactis praelongus*, endodermal muscles are directly photosensitive and responsive to light (Marks 1976). Light sensitivity in cells that are not considered neurons suggests that cnidarians could be using opsins in unconventional ways, either by sensing light with non-neuronal cells or by using opsins for roles other than light sensing.

**Fig. 1:**
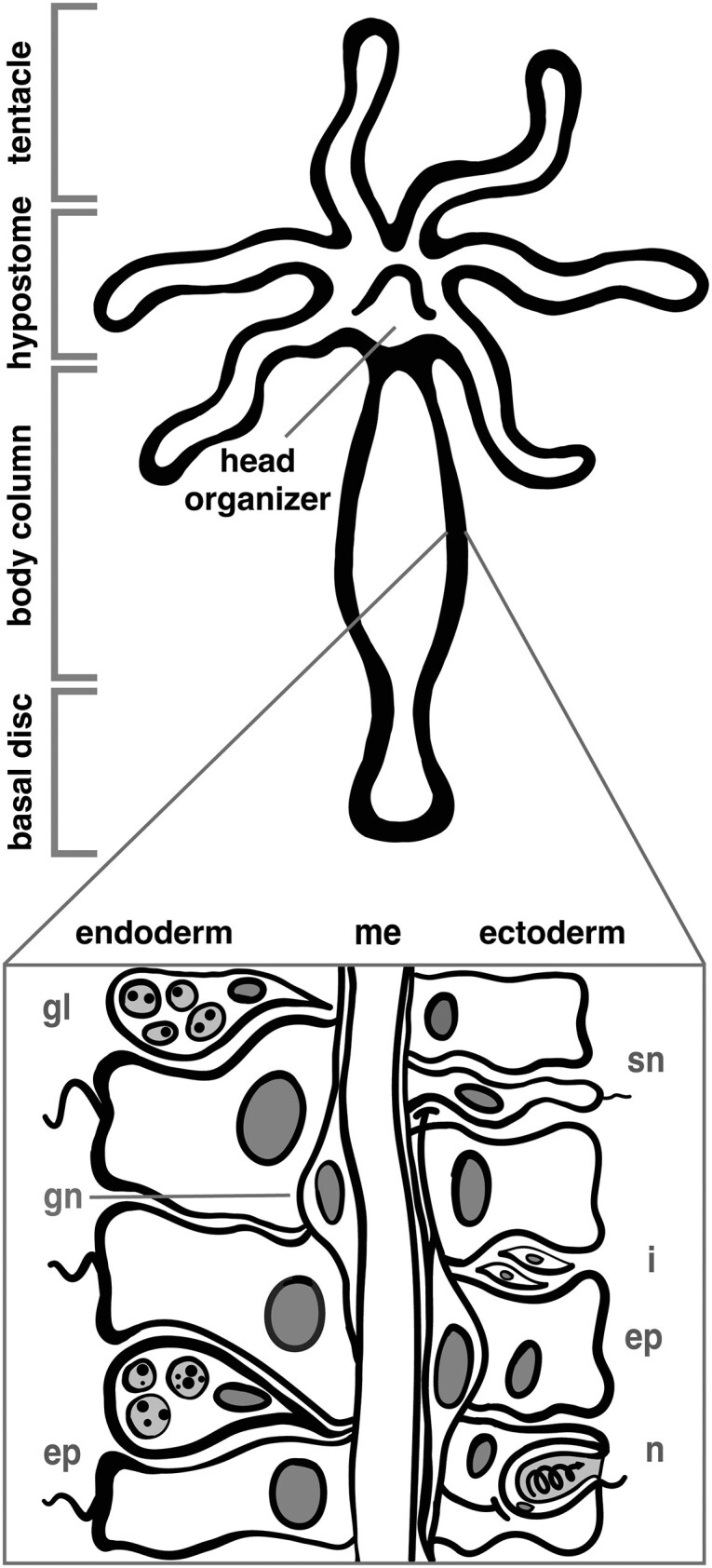
*Hydra* body plan and cell types. Here is a diagram of the *Hydra* body plan to show the location of the basal disc, body column, hypostome, and tentacle and denote the ectodermal and endodermal cell layers in *Hydra*. In the endodermal layer, there are gland cells (gl), ganglion neurons (gn), and epithelial cells (ep). In the ectoderm there are sensory neurons (sn), interstitial stem cells (i), epithelial cells (ep), and nematocytes (n). The two cell layers are separated by an extracellular matrix known as mesoglea (me). Adapted from Technau and Steele (2011) and Siebert et al. (2019).

To begin exploring the opsin gene family’s involvement in non-neuronal photosensitivity, a first step is to establish what types of cells express opsins, ideally in a well-characterized model organism to facilitate future functional studies. Here, we used previously published data to investigate the expression patterns of opsin genes across cell types in *Hydra vulgaris*, a small (5–20 mm) freshwater cnidarian model with a simple body plan, high-quality publicly available genomic and transcriptomic datasets, and well-characterized cell types. *Hydra* can sense and respond to light despite lacking distinct photosensory structures (Hunt et al. 2014; Guertin and Kass-Simon 2015). Hydra has three main cell lineages: interstitial, ectodermal epithelial, and endodermal epithelial (Bosch et al. 2010). Each of these lineages arises from their own adult stem cell populations that maintain the organism by replacing aging and damaged cells (Bosch et al. 2010). Stinging cells (nematocytes), neurons (sensory and ganglion), and gland cells all arise from the multipotent interstitial stem cell lineage, while epithelial cells arise from the unipotent ectodermal and endodermal stem cell lineages (Fig. 1). *Hydra* also have a group of cells in the apical region known as the head organizer, which signal to neighboring cells to differentiate into hypostome and tentacles through the expression of developmental patterning genes. This region also plays key roles in regeneration and reaggregation after injury (Broun and Bode 2002) (Fig. 1).

By using single-cell data (24,985 cells) and trajectory inference methods from the landmark scRNA-seq study on cell differentiation trajectories in *H. vulgaris* by Siebert et al. (2019), we find that cells across all three lineages (interstitial, ectodermal, and endodermal) express opsins. Opsins are expressed in both neuronal and non-neuronal cell types of the interstitial stem cell lineage and cell types deriving from entirely different stem cell lineages, such as ectoderm and endoderm. While neuronal cell type clusters expressed a greater diversity of opsin transcripts, non-neuronal cell type clusters, in general, exhibited fewer distinct transcripts with a higher percentage of cells in the cluster expressing them. Some opsins found in the datasets also shared enriched transcription factor (TF) binding motifs with genes associated with development and patterning. By characterizing the expression patterns and regulatory associations of genes beyond our common conception of their gene family’s function, we can begin filling the gaps formed by our traditional associations between genes and cell type function. This can help to strengthen the foundation on which we begin to understand how cell types evolve and diversify.

## Materials and methods

### Single cell dataset

We used the single-cell RNA sequencing data generated by Siebert et. al. (2019), which characterized the molecular diversity of cell types in *H. vulgaris* (available from Dryad https://doi.org/10.5061/dryad.v5r6077). This dataset encompasses 24,985 single-cell transcriptomes produced with Drop-seq (Macosko et al. 2015), from a range of cell types and differentiation states of whole *H. vulgaris*.

### Identifying opsin transcripts

**Fig. 2:**
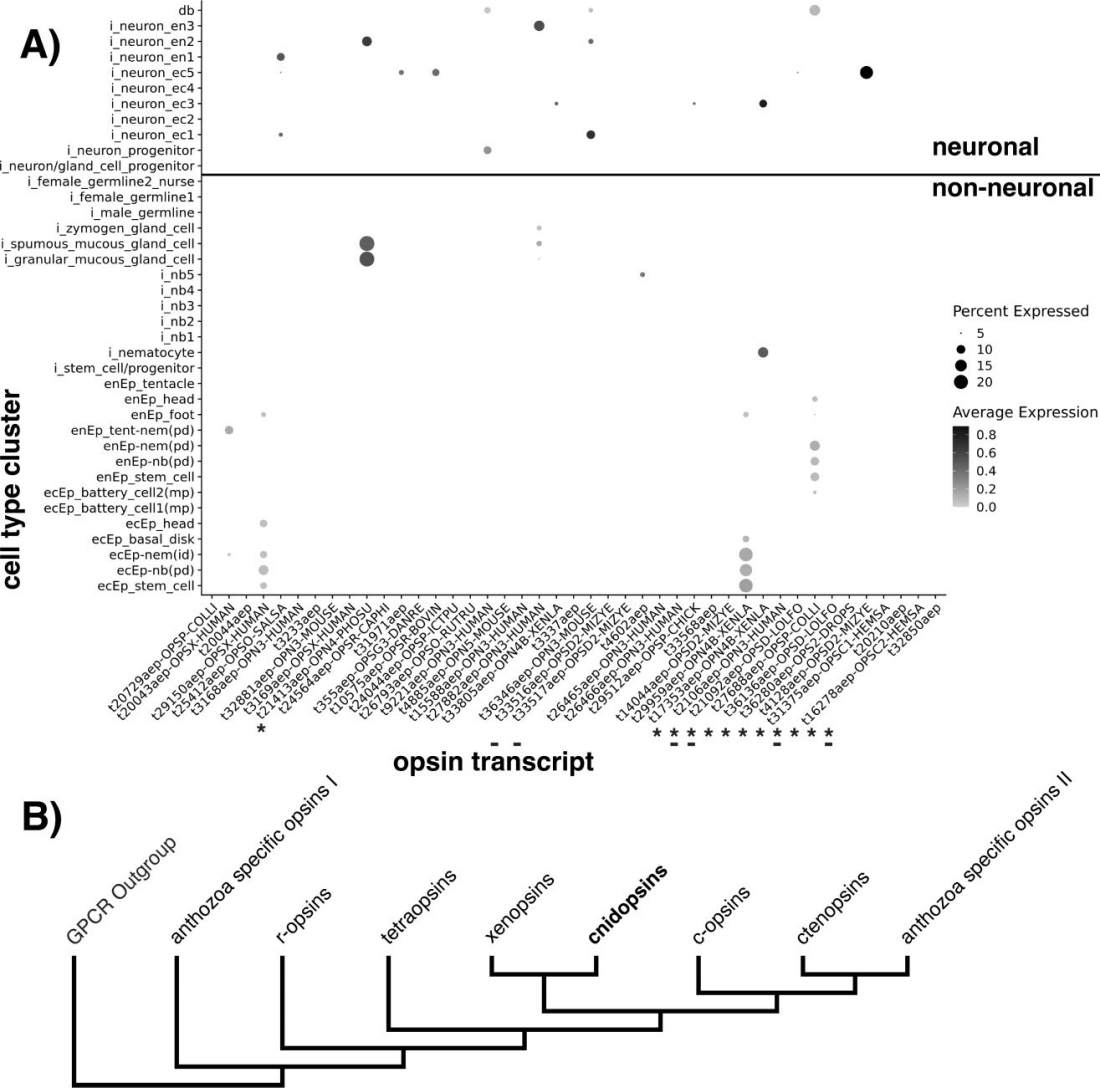
(A) Opsin gene expression across cell types in *Hydra*: The *x*-axis denotes all of the opsin gene transcripts found in the *Hydra* dataset and the *y*-axis denotes all of the cell type categories identified in the *Hydra* dataset. The size of the dot denotes the percent of the cell type cluster the opsin was found expressed in (minimum 5% expression cutoff, dot size scaled by radius) while the shade denotes the average expression (log1p normalized UMI counts) of the opsin across the whole cluster. The cell types are first divided by lineage, “i” meaning interstitial, “en” meaning endoderm, and “ec” meaning ectoderm, and then the cell type is denoted. From Siebert et al. (2019): ec, ectodermal; en, endodermal; Ep, epithelial cell; gc, gland cell; id, integration doublet; mp, multiplet; nb, nematoblast; nem, differentiated nematocyte; pd, suspected phagocytosis doublet; prog, progenitor. id, mp, and pd are categories of biological doublets. All transcripts marked with a “*” under the ID were fragmented and all transcripts marked with a “-” did not have sites of open chromatin, as denoted in Table 1. (B) Simplified opsin tree: Here, we show a cladogram depicting the relation of the cnidopsins clade (containing opsins from *Hydra* and other cnidarians) to other major opsin clades. (Adapted from Gornik et al. 2021).

We identified opsin transcripts in the low redundancy reference *H. vulgaris* AEP transcriptome (38,749 sequences) from Siebert et al. (2019) using a command line version of Phylogenetically Informed Annotation (PIA) (Speiser et al. 2014). PIA allows rapid phylogeny-informed annotation rather than simply using sequence similarity. The pipeline first uses TransDecoder (v5.5) to infer the longest open reading frames from each transcript, then identifies candidate genes using BLASTp to a set of opsin proteins from diverse taxa with an e-value threshold of 1e-10. Next, the pipeline uses MAFFT (v7.480) to add candidate opsins to a precalculated opsin alignment and then RAxML (v8.2.12) to place candidate opsins in a precalculated opsin tree (both alignment and tree from Picciani et al. (2018)). After retrieving opsins from the AEP transcriptome using PIA, we performed an additional phylogenetic analysis in order to infer their relationships with other medusozoan opsins, especially *Hydra* opsins from Macias-Muñoz et al. (2019) and opsins retrieved from the protein models in the *Hydra* 2 Genome Project using PIA. To improve taxonomic diversity of medusozoan opsins, we added new sequences that were not included in Picciani et al. (2018). We again used PIA to retrieve cnidops sequences from genome-based protein models from *C. hemisphaerica* (10 sequences), *Morbakka virulenta* (2 sequences), *Rhopilema esculentum* (6 sequences), *Alatina alata* (6 sequences), *Hydractinia symbiolongicarpus* (10 sequences), and *Calvadosia cruxmelitensis* (15 sequences). We combined these new 49 cnidops sequences with 177 cnidops sequences from Picciani et al. (2018) and other 133 cnidops sequences from the *Hydra* AEP low redundancy transcriptome, Macias-Muñoz et al. (2019) and the *Hydra* v2 protein models. We aligned them using the MAFFT E-INS-I algorithm and inferred a maximum-likelihood tree with IQTREE2, with model LG + F + R10, and 1000 ultrafast bootstrap replicates (Supplementary Files 1 and 2). A simplified version of the opsin gene tree is illustrated in Fig. 2(B) to show the relation of the cnidopsins to the rest of the clade (Gornik et al. 2021).

### Plotting opsin expression across cell types and states in *Hydra*

**Fig. 3:**
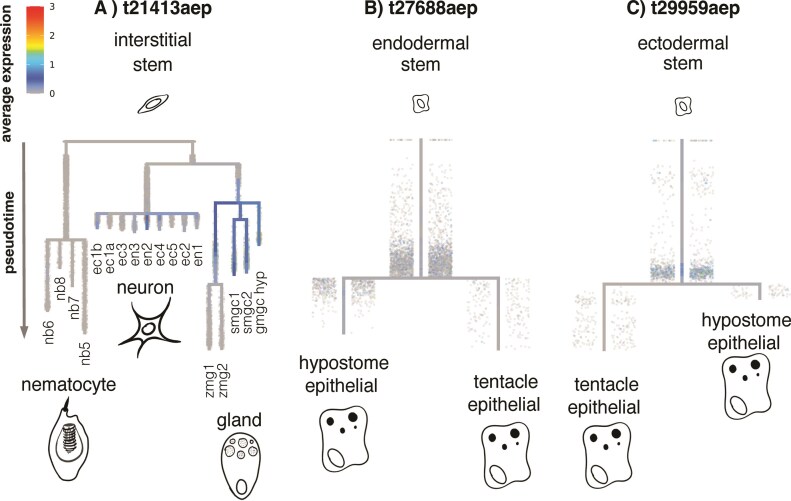
Dendrograms of overall gene expression changes in pseudotime colored by opsin expression in the three cell lineages in *Hydra*: These dendrograms are plotting the expression of (A) t21413aep in the interstitial lineage, (B) t27688aep in the endodermal lineage, and (C) t29959aep in the ectodermal lineage. Each cell in the lineage is denoted by a dot, the color represents expression level with red indicating higher expression than blue. Branch length in the plot denotes pseudotime as cells progress through differentiation. Here, we see t21413aep detected in neuronal and gland cell types while t29959aep and t27688aep are detected in cell types just before differentiation to tentacle or hypostome ectodermal and endodermal cells, respectively.

Opsin expression across the *Hydra* single cell dataset was plotted using the URD *Hydra* Plotting tutorial (Farrell et al. 2018) in a Jupyter Notebook. First, we produced a dot plot using Seurat (v3), where the *x*-axis represents all the opsins found in the low redundancy reference transcriptome, and the *y*-axis represents the cell type clusters inferred by Seibert et al. (2019) (Fig. 2A). Transcripts t37969aep and t24989aep were not in the URD objects and so are not in the expression plots, likely because they were too fragmented or lowly expressed. URD is a package for reconstructing cell differentiation trajectories based on gene expression​. URD simulates reconstruction using single-cell RNA seq data and is an unobserved, non-deterministic measure of a cell’s transition through cell states. Gene expression is plotted over “pseudotime” rather than set time points (Reid and Wernisch 2016). Cell state refers to cells of a common phase in differentiation (stem, progenitor, and so on), while cell type refers to cells of a common type when clustered by cell marker expression (neuron, nematoblast, ectoderm, and so on). The R objects from the Seibert et al. (2019) URD analyses were loaded from Dryad, and each opsin transcript was plotted on a dendrogram for each of the three stem cell lineages (interstitial, ectoderm, and endoderm) (Fig. 3 and Supplementary File 3). The branch length on the dendrogram denotes pseudotime and dot color denotes expression level. For this paper, opsin expression in neuronal cell types is defined as expression detected in neuron cell type clusters and neuron progenitor clusters. Expression in cell-type clusters that are not neurons or neuron progenitors is considered opsin expression in non-neuronal cell types.

**Fig. 4:**
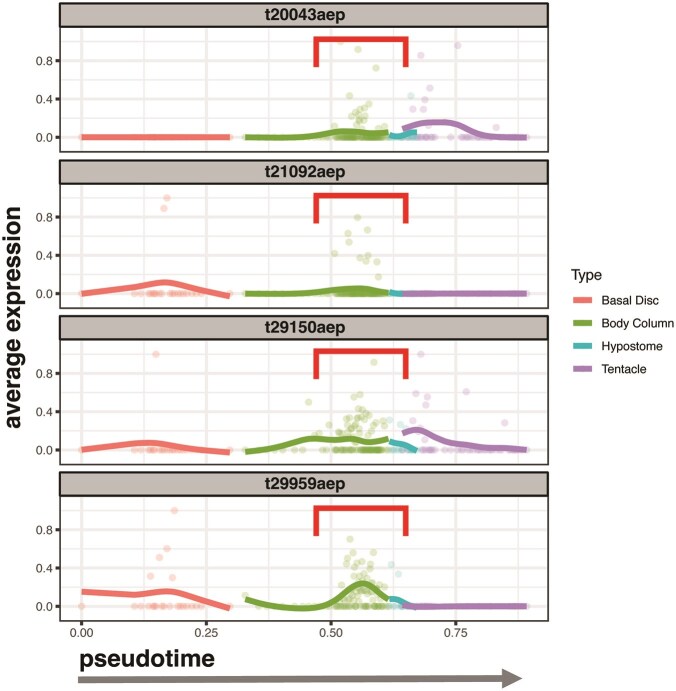
Multi-segment spline plot of opsin expression in ectodermal cells: This multi-segment spline plot shows the expression of four opsin genes (t20043aep, t21092aep, t29150aep, and t29959aep) over pseudotime, with spatially relevant cell type groups (basal disc, body column, hypostome, and tentacle) annotated as colored line segments. The peak in expression is seen between the body column and hypostome, which potentially indicates opsin expression in the head organizer (red bracket).

From these plots, we pinpointed in which cell types and when during their differentiation opsins were more highly expressed. We specifically looked for patterns such as expression in neuronal cell types (neuron and neuron progenitors) versus non-neuronal cell types (nematoblast, granular, ectodermal, and endodermal), expression in multiple cell types of the same lineage (meaning they differentiate from the same stem cell population), or expression in multiple cell states of the same lineage (Figs. 1 and 2; Supplementary File 3). An opsin transcript was considered expressed when it was detected in greater than 5% of cells in a given cell type cluster (Fig. 2A). Opsins are G protein-coupled receptors (GPCRs) and as such can initiate meaningful signals even at low expression levels due to amplification during G protein-mediated signal transduction cascades (Arshavsky and Burns 2014). In Supplementary File 3, we also include two versions of Fig. 2(A) using a 10% and a 0.01% expression cutoff, respectively. We plotted transcripts with noticeably higher levels of expression across cell states from the ectodermal lineage, on multi-segment spline plots, which visualized expression over pseudotime (Fig. 4). The multi-segment spline plot allows for easier estimation of changes in expression than the dendrogram because the expression is plotted on the *y*-axis rather than as a color gradient. The spline is split into segments by spatially relevant regions in the oral-aboral axis (basal disc, body column, hypostome, and tentacle) based on marker gene expression validated in Seibert et al. (2019) through RNA *in situ* hybridization (RNA-ISH).

### Identification and localization of increased transcriptional activity on opsin genes

We made a table of sites of increased transcriptional activity near each opsin locus, based on data from the Assay for Transposase-Accessible Chromatin (ATAC-Seq) in the Hydra 2.0 Genome Project Portal (HGP) (Chapman et al. 2010; Siebert et al. 2019; “Hydra 2.0 Web Portal” 2025). ATAC-seq targets areas of open chromatin where increased transcriptional activity is likely occurring, and this allows us to infer which opsins have increased regulatory and transcriptional activity and where this occurs on the gene locus. To find the opsin genes in the Hydra 2.0 Genome Project Portal ATAC-seq tracks (whole-animal, replicates 1–3), we blasted the opsin proteins against the genome to retrieve scaffolding IDs. Using this information, we recorded the ATAC- seq peaks on or near each opsin gene (Table 1). Any peak found 10 kb up- or downstream of the start or end of a transcript were recorded in the table with (US) denoting upstream, (IN), denoting intronic, and (DS) denoting downstream. Previous studies using ATAC-seq data in *Hydra* have used smaller windows (2–5 kb from the transcription start site [TSS]) (Siebert et al. 2019; Murad et al. 2021), but more recent studies have suggested that while the majority of conserved regulatory elements do fall within 2 kb of the locus, there is evidence for distal enhancer activity in *Hydra* (Reddy et al. 2020; Cazet et al. 2023). For this reason, we use a larger window to capture the proximal promoter region generally found within 2 kb of the TSS in addition to more distant enhancers between gene loci. Direction was identified from annotation of the Augustus gene modules, so if the Augustus gene module was missing (i.e., * fragmented sequences in Table 1), the location of the peak was denoted as (?) (Table 1). Augustus gene modules found in tandem on the (HGP) were denoted as tandem groups (Supplement 4).

**Table 1:** Opsin transcripts found in the low-redundancy transcriptome assembly from *H. vulgaris* strain AEP by Siebert et al. (2019)

Transcript ID	Augustus gene module from HGP	ATAC seq peak number	Metagene
t20729aep	Sc4wPfr_704.g18853.t1	64,499 (DS) 64,498 (US)	wg23
t20043aep	Sc4wPfr_558.1.g10186.t1	56063, 56064, 56065, 56067 (US) 56062, 56061 (DS)	wg30
t20044aep	Sc4wPfr_558.1.g10186.t1	56063, 56064, 56065, 56067 (US) 56062, 56061 (DS)	wg30
t29150aep	Sc4wPfr_558.1.g10185.t1	56055, 56056 (US)	wg17
t25412aep	Sc4wPfr_363.3.g30488.t1	40344, 40345 (DS)	wg63
t3168aep	Sc4wPfr_338.g25858.t1	37598 (DS) 37599 (IN) 37001, 37601, 37601 (US)	wg50
t3233aep	Sc4wPfr_338.g25854.t1	37575 (US) 37577, 37578, 37579, 37580, 37581 (IN) 37582 (DS)	–
t32881aep	Sc4wPfr_338.g25854.t1	37575 (US) 37577, 37578, 37579, 37580, 37581 (IN) 37582 (DS)	–
t3169aep	Sc4wPfr_338.g25854.t1	37575 (US) 37577, 37578, 37579, 37580, 37581 (IN) 37582 (DS)	–
t21413aep	Sc4wPfr_303.g13691.t1	34163, 34164, 34165 (DS) 34166 (US)	wg76
t24564aep	Sc4wPfr_200.g3548.t1	21480, 21481 (DS) 1482 (US)	–
t31971aep	Sc4wPfr_200.g3547.t1	21476, 21477, 21478 (US) 21479 (DS)	wg29
t355aep	Sc4wPfr_200.g3547.t1	21476, 21477, 21478 (US) 21479 (DS)	wg29
t10575aep	Sc4wPfr_200.g3546.t1	21473, 21474, 21475 (US) 21476, 21477, 21478 (IN) 21479 (DS)	wg29
t24044aep	Sc4wPfr_200.g3536.t1	21461 (US) 21462, 21463 (DS)	–
t26793aep	Sc4wPfr_200.g3518.t1	21409, 21410, 21411 (US)	–
t9221aep	Sc4wPfr_199.g28613.t1	21005 (DS)	–
t4885aep	Sc4wPfr_199.g28610.t1	20994 (US)	wg22
t15588aep	Sc4wPfr_199.g28610.t1	20994 (US)	wg22
t24989aep	Sc4wPfr_199.g28609.t1	20992 (DS) 20993 (US)	–
t27882aep	Sc4wPfr_199.g28609.t1	20992 (DS) 20993 (US)	–
t33805aep	Sc4wPfr_172.1.g1616.t1	17064, 17065, 17066 (US) 17063 (DS)	wg23
t37969aep	Sc4wPfr_17.g15899.t1	16739, 16740, 16741, 16742, 16743 (DS)	–
t3337aep	Sc4wPfr_17.g15864.t1	16653 (US) 16654, 16655 (IN)	–
t36346aep	Sc4wPfr_17.g15863.t1	16646, 16647, 16648 (US) 16649, 16650 (DS)	wg63
t33516aep	Sc4wPfr_163.g17463.t2	–	–
t33517aep	Sc4wPfr_163.g17463.t1	–	wg44
t4602aep	Sc4wPfr_161.g25557.t1	14854, 14855, 14856, 14857 (US) 14858, 14859, 14860 (DS)	wg16
t26465aep	Sc4wPfr_161.g25553.t1	14844 US 14841, 14842, 14843 (IN) 14840 (DS)	–
t26466aep	Sc4wPfr_161.g25553.t1	14844 US 14841, 14842, 14843 (IN) 14840 (DS)	–
t29512aep	Sc4wPfr_126.g26211.t1	8408 (US) 8408 (DS)	wg55
t33568aep	Sc4wPfr_126.2.g29332.t1	8723 (US) 8724, 8725 (DS)	wg21
t14044aep	Sc4wPfr_126.2.g29332.t1	8723 (US) 8724, 8725 (DS)	wg21
t29959aep	Sc4wPfr_1241.g21488.t1	8098, 8097, 8096, 8095, 8094 (US) 8093, 8092, 8091 (DS)	wg17
t17353aep	*	12391, 12392 (?)	–
t2106aep	*	–	–
t21092aep	*	–	–
t27688aep	*	23571, 23572 (?)	–
t36136aep	*	12443 (?)	–
t36280aep	*	38355 (?) 3856 (?)	–
t4128aep	*	16720 (?) 16721, 16722, 16733 (?)	–
t32850aep	*	–	–
t31375aep	*	–	–
t20210aep	*	16658, 16659 (?) 16657 (?)	–
t16278aep	*	1497, 1498 (?)	–

This table contains the opsin transcripts identified with PIA, the Augustus gene module ID retrieved from the Hydra 2.0 Genome Project Portal, the peak number and location from the ATAC-seq tracks from the Hydra 2.0 Genome Project Portal, and metagenes (as inferred by Siebert et al. 2019). Peak numbers followed by (US) denote peaks upstream from the genome locus, while (DS) denotes peaks downstream, and (IN) means intronic, when there is no gene locus to orient direction (?) is used for peak localization. Rows are ordered by gene ID, so when transcripts align to the same gene locus, the gene ID cells are merged. Some opsin genes were not associated with any metagene, and some transcripts detected were too fragmented to associate with a gene ID (*) with which to reference in the NMF analysis performed by Siebert et al. (2019).

### Identification of genes co-regulated with opsins

Siebert et al (2019) used non-negative matrix factorization to place genes into “metagenes,” groups of co-expressed genes that, in some cases, were associated with significantly enriched TF binding motifs. The metagene with the highest correlation score for each opsin found in the dataset is recorded alongside the ATAC-seq peak annotations(Table 1). Co-expressed genes found in the same metagenes as the opsins found in the dataset were noted and their annotations were referenced from UniProt (Supplement 4).

## Results

### Opsin diversity in the *H. vulgaris* AEP transcriptome

We identified 45 opsin transcripts (with two transcripts possibly being the ends of the same transcript) in the low-redundancy *H. vulgaris* AEP transcriptome (Siebert et al. 2019), which aligns with previous studies (Table 1 and Supplementary File 1). The first studies in *Hydra* detected 63 full-length opsin gene sequences, whereas more recent studies using more contiguous genomes detected closer to 45 (Plachetzki et al. 2007; Suga et al. 2008; Macias-Muñoz et al. 2019). While the number of opsin transcripts we found matches the number of opsin genes found in other studies, we note that in many cases, we find multiple transcripts expressed from the same gene locus (Table 1). Of the opsin gene loci detected in the HGP, six were potentially generating multiple isoforms (2–3) (Table 1). This means the number of opsin genes we detected is likely different from those found in other studies.

Our phylogenetic analysis places all of the 45 opsin transcripts in the canonical type-2 animal opsin clade (Supplementary File 2), within the xenopsins (Ramirez et al. 2016) and in the cnidops clade (Plachetzki et al. 2007). We corroborate most phylogenetic results from Macias-Muñoz et al. (2019) and retrieve clades B, C, and D (Supplementary File 1). Fourteen out of these 45 opsin transcripts are fragmented, ending before the lysine residue position. Where known, all opsins in clades C and D contain the conserved lysine residue for retinal binding, often considered diagnostic for opsins, with two exceptions (t25412aep and t37969aep). The clade B opsin OpB1 (t21413aep) lacks the conserved lysine but is nested far within other opsins and contains conserved domains of type-2 opsins, so we annotate it as an opsin and consider the absence of lysine to be a secondary loss. While Macias-Muñoz et al. (2019) annotated t29274aep (corresponding to *HvOpA1*) as an opsin, here we found that it does not belong to the clade with type-2 opsins and does not contain the critical lysine, so we do not annotate it as an opsin (Table 1 and Supplementary File 2). Recently, De Vivo et al. 2023 highlighted the existence of a group of opsin-related sequences, or “pseudopsins,” that are closely related to placozoan opsins (placopsins) (Feuda et al. 2012) in a possible sister clade to type-2 animal opsins. We recovered many possible *Hydra* pseudopsins closely related to OpA1 (t29274aep), other cnidarian pseudopsins, and placopsins (Supplementary File 2). Based on their phylogenetic position, these pseudopsins are speculated to be capable of acting as GPCRs but lack the lysine residue so may not be light sensitive. In this study, we restrict our analysis of opsin expression across *Hydra* cell types to canonical type-2 animal opsins.

#### Neuronal cell types express a wider diversity of opsins than other cell types, mostly in their differentiated cell states

We detected 13 different opsin transcripts in various neuronal cell types, often with multiple transcripts in a single cell-type cluster (Fig. 2A). Among neuronal cell types expressing opsins (neuron progenitors, ectodermal neuron clusters 1, 3, and 5, and endodermal neuron clusters 1–3), between 5% and ∼20% of the cells in a cluster expressed at least one opsin transcript (Fig. 2A). In the neuronal progenitor cell cluster, only one opsin transcript (t9221aep) was detected per cluster, and was expressed in only ∼5% of cells. In differentiated neuronal cells, cell clusters often expressed multiple different opsin transcripts (∼2–4 at most 5) with a higher percentage of cells in their respective cluster expressing each of those transcripts relative to the one transcript found in the neuron progenitor cluster. Only one transcript (t9221aep) was found both in progenitor and fully differentiated neuronal cell types (Fig. 2A).

#### Non-neuronal cell types express fewer opsin transcripts than neuronal cell types, but do so at a higher percent expression across more cell states

For non-neuronal cell types, fewer opsin transcripts were detected, primarily in gland cells and nematocytes (Fig. 2A). Each cell type cluster generally had 1–2 transcripts (3 at most) in up to 20% of the cells in their respective cluster (Fig. 2A). Additionally, we find that t21413aep (ortholog of OpB1 from Macias-Muñoz et al. 2019), an opsin lacking the lysine residue, is highly expressed during the differentiation trajectory of gland cells. This opsin is expressed in the fully differentiated states of spumous mucous gland cells and granular mucous gland cells, and in their progenitors shared with zymogen gland cells (Fig. 3A).

Fully differentiated gland cells and nematocyte clusters also tended to express the same transcripts as their earlier cell states (Fig. 2A). For example, transcripts t29150aep and t29959aep were detected in the ectodermal epithelial lineage throughout cell state transitions (Fig. 2A). These transcripts were found in the ectodermal epithelial stem cells, ectodermal epithelial nematoblasts, and ectodermal epithelial nematocytes following the differentiation trajectory expected for nematocytes (Fig. 2A). Transcript t27688aep showed the same expression patterns but in endodermal epithelial stem cells, endodermal epithelial nematoblasts, and endodermal epithelial nematocytes (Fig. 2A). In the dendrogram plots, t27688aep also shows expression right at the differentiation point between the two endodermal epithelial cell lineages (Fig. 3B)

We also found t27688aep (no 1:1 ortholog), t20043aep (OpD31), t4602aep (OpD10), and t17353aep (OpD21) all expressed in nematocytes and nematoblasts even though some were not recorded as such by Macias-Muñoz et al. (2019). This can be due to the differences in the threshold by which we define expression but most likely due to orthology inference between opsin sequences from different *H. vulgaris* strains (*Hydra* 105 in Macias-Muñoz et al. 2019 and *Hydra* AEP in Siebert et al. 2019). We also noted that many of these transcripts were putative isoforms from the same gene, but showed differing expression patterns.

#### Some opsin transcripts exhibited peaks in expression in ectodermal cells between the body column and hypostome

Four transcripts in particular (t20043aep, t21092aep, t29150aep, and t29959aep) showed noticeably higher expression levels in the ectodermal lineage dendrogram plots (Fig. 3C; Supplementary File 3). Their expression peaked at the split from ectodermal progenitors to two differentiated ectodermal cell clusters for the tentacle and hypostome (Fig. 3C). This pattern was also exhibited when the transcripts were plotted on multi-segment spline plots (Fig. 4). Transcripts t29150aep and t29959aep show an increase in expression in the portion of the body column nearest the hypostome, even more so than t20043aep and t21092aep (Fig. 4). This peak correlates to where the head organizer should be found on the *Hydra* body plan (Fig. 1).

#### 39 out of 45 opsin transcripts exhibited open chromatin sites around their locus

Thirty-nine of the 45 opsin transcripts we identified had open chromatin regions upstream, downstream, or within their genomic locus (Table 1). In many of these cases, there were multiple peaks of open chromatin, which may indicate high transcription activity in and around these opsins (Table 1). Many of these sites of high transcriptional activity were associated with opsins expressed in non-neuronal cell types (gland cells, nematocytes, and their progenitor clusters) (Table 1 and Fig. 2A). We also find that all opsins expressed in neuronal and non-neuronal cells had open chromatin sites with 10 kb (Fig. 2A and Table 1).

#### 20 opsin transcripts were members of metagenes, three of which were enriched with TF binding motifs

Siebert et. al. (2019) used non-negative matrix factorization to define groups of co-expressed genes (metagenes) and identify associated enriched TF binding motifs. We found 20 opsin transcripts in 12 distinct metagenes, with multiple metagenes containing more than one opsin (Table 1). Three of these metagenes (wg17, wg44, and wg76) were enriched with binding motifs for TFs involved in development, such as Six3/6, Otx, Dlx, Rfx4, and HoxA (Table 1 and Table S4).

## Discussion

In this study, we characterize the expression pattern of 45 opsin transcripts across all three cell lineages in *Hydra*, highlighting distinct patterns of expression in non-neuronal cells such as gland cells and nematocytes. Many of these opsins expressed in non-neuronal cells have multiple peaks of open chromatin up and downstream of the gene locus indicating strong regulatory activity, are associated with multiple regulatory motifs involved in development, and peak in expression in key developmental patterning regions of *Hydra*. Taken together, these results indicate that opsins may play a significant role in the differentiation and functioning of gland cells and nematocytes in *Hydra*. In order to more concretely understand these roles, future studies can disentangle the contribution of individual opsin genes through heterologous expression, and knockdowns and knockouts; coupled with developmental and behavioral assays.

### Neuronal subtype clusters with distinct repertoires of opsin expression could indicate distinct functions

The opsin gene family has a well-documented history of gene duplications and functional differences, and cnidarian opsins are no exception (Plachetzki et al. 2007; Suga et al. 2008; Liegertová et al. 2015; Picciani et al. 2018; Macias-Muñoz et al. 2019; Gornik et al. 2021). One way opsins might differ in function is through the mutation of sequences in spectral tuning sites, which influence the wavelengths of light a given opsin receptor can detect (Yokoyama et al. 2008; Rajamani et al. 2011). Given that neuron sub-populations in *Hydra* can be transcriptionally and functionally distinct and we find these neuron subclusters each express multiple opsin transcripts to differing degrees, if these opsin transcripts are translated into functional proteins, we may see wavelength-specific responses to light among distinct neuron sub-populations (Dupre and Yuste 2017; Siebert et al. 2019). Previous studies on the wavelength dependence of cnidarian larval swimming behavior further corroborate the possibility of wavelength-dependent behavior in cnidarians (Lilly et al. 2024). New computational methods for predicting lambda max values from opsin gene sequences (Frazer et al. 2024) could be one way to begin investigating the wavelength specificity of these opsins in *Hydra*. There can also be non-wavelength related explanations for neuron sub-populations having distinct expression patterns such as optimizing for light intensity sensitivity, or simply non-adaptive retention of expression of duplicated genes. Ultimately, functional or behavioral assessment of opsins in neuronal cell types is necessary to make any firm claims about organismal function.

### Separate opsin genes and putative isoforms show distinct expression patterns in nematocytes of *Hydra*

Opsin t17353aep (found in interstitial nematocytes) and t29959aep (found in ectodermal epithelial nematoblasts and nematocytes) share the same gene ID, meaning they are potentially isoforms of the same gene (Table 1). This shows that different putative isoforms from the same gene can be found in the same broad cell type category but can be expressed in different subtype clusters. In addition, Opsin t20043aep (found in endodermal epithelial nematoblast and nematocytes) and t29150aep (found in ectodermal epithelial nematoblast and nematocytes) come from two different genes (Sc4wPfr_558.1.g10186.t1 and Sc4wPfr_558.1.g10185.t1), which are found in tandem on the *Hydra* genome and could represent a tandem duplication and then a change in expression regulation to different cell type sub-clusters (Chapman et al. 2010).

### Revisiting light-mediated nematocyte discharge in *Hydra*

Light influences the firing of nematocytes in battery complexes in *Hydra*, probably via opsin-mediated phototransduction in adjacent neurons (Plachetzki et al. 2012). Based on *in situ* hybridization, opsin HmOp2 co-localized with two key phototransduction elements, cyclic nucleotide-gated ion channel (CNG) and Arrestin, in the sensory neurons of battery complexes, but not expressed in nematocytes. Here, we find t29512aep (within the same clade as OpD5, which was identified as HmOp2 by Macias-Muñoz et al. (2019) [Supplementary File 1]) to only be expressed in neurons (Fig. 2A). This indicates that while one opsin may be specific to neurons in battery complexes, there are still others found in this study that may be expressed in nematocytes of battery complexes for opsin-mediated phototransduction. Interestingly, when the *Hydra* phototransduction cascade elements identified in Macias-Muñoz et al. (2019) are plotted in a dot plot similar to Fig. 2(A), CNG (t27655aep) is not found expressed in any cells, and Arrestin (t14420aep) is found across many cells (Supplementary File 3). There were many other phototransduction cascade elements found highly expressed in cells found by this study to express opsins (including nematocytes), which is a positive sign that these opsins may be capable of mediating phototransduction (Supplementary  File 3).

### Gene expression and regulatory patterns suggest potential developmental roles for opsins in non-neuronal cell types in *Hydra*

In nematocytes, some opsin transcripts (t29150aep, t29959aep, and t27688aep) were expressed throughout early and later cell states (Fig. 2A). The expression of opsins in the ectodermal epithelial stem cell, ectodermal epithelial nematoblast, and ectodermal epithelial nematocytes could indicate these cells using light as a factor in processes such as differentiation or migration. Given that opsins and light sensitivity can play a role in cell fate decisions for keratinocytes (Castellano-Pellicena et al. 2019), this would not be unprecedented. As for cell migration, Siebert et al. (2019) showed that other transcripts were expressed in a position-dependent manner as cells migrated along the *Hydra* body column. As nematocytes differentiate and migrate through the ectodermal cell layer of the body column, they may require some external cue, such as light, to indicate their position (Campbell and Marcum 1980). Our findings of multiple opsin transcripts expressed across nematocyte cell state transitions may indicate that this process is influenced by opsin-mediated phototransduction. As mentioned before, we do also find some genes proposed as phototransduction cascade elements in *Hydra* to be expressed alongside these opsins in early and later states of nematocytes (Supplementary File 3).

The expression of opsin transcripts in cells near the head organizer could also suggest a role for opsins in cells transitioning through states of differentiation. The transcripts t29150aep, t29959aep, t20043aep, and t21092aep all showed increased expression levels in ectodermal cells in a region of the body column close to the hypostome. As mentioned before, these spatially relevant body regions were reconstructed by URD on multi-segment spline plots and validated with RNA-ISH of marker genes in Siebert et al. (2019). This region where the opsins were expressed is near the head organizer, which plays important roles in axial patterning and regeneration via signaling to neighboring cells to differentiate through the expression of developmental patterning genes (Broun and Bode 2002; Bode 2003). Association with the head organizer is further supported by t29150aep and t29959aep sharing regulatory factors with a shared metagene driven by TFs associated with developmental patterning and regeneration. Wg17 was enriched in factor binding motifs that bind to CRX, Ptx1, oc, Crem, XBP1, ATF7, Arx, and Alx4 in other animals, according to the JASPAR database (Khan et al. 2018). According to JASPAR and PFAM matches listed in Table S5 from Siebert et al. (2019), these motifs are cis-regulatory elements that, in *Hydra*, are likely to bind key TFs such as Six3/6, which in cnidarians is known to regulate aboral patterning (Sinigaglia et al. 2013), Otx which plays a role in axis formation in the head organizer (Simeone et al. 1993; Suda et al. 1999), Dlx which functions in anterior patterning (Asano et al. 1992), Rfx4 which is expressed in gland cells in the apical sensory organ of *Nematostella* larvae (Gilbert et al. 2022), and HoxA which provides cells with temporal and spatial positional identities on the anterior-posterior axis (Lee et al. 2006). The co-regulatory relationship between opsins and developmental patterning genes in early and later cell states near the head organizer indicates a possible influence of light on development, cell migration, and regeneration in *Hydra*. These findings invite further investigation with functional assays testing for modulation of cell proliferation, cell migration, and regeneration under varying light conditions.

### Opsins in *Hydra* can serve as a model system to investigate the underlying mechanisms of light-mediated processes in Cnidaria

Many behavioral and physiological processes may be mediated by circadian rhythms queued by light/dark cycles in Cnidaria (i.e., vertical diel migration and neurotransmitter regulation). Recently, there has been strong progress in establishing the molecular basis for circadian rhythms in cnidarian models such as *Nematostella*, but there is still room to expand our understanding of which behaviors and physiologies are directly influenced by light, through what molecular pathways, and in what cell types (Berger and Tarrant 2023; Aguillon et al. 2024). Berger et. al. (2023) find that locomotor and transcriptional rhythms are disrupted when light and temperature cycles are misaligned, while metabolic rhythms are retained. Cycles of behavioral and transcriptional day-night cycles have also been observed in *Hydra* despite the absence of canonical clock genes in their transcriptome analyses, but finer detail into the dynamics of disruptions of this cycle may be needed (Kanaya et al. 2019). By investigating the regulatory networks shared by the opsins found in this project and genes known to vary in diurnal cycles, and by further characterizing these expression patterns in other cnidarian and metazoan models through comparative analyses, we can further piece together the origin and evolution of mechanisms driving circadian rhythms.

## Conclusion

As sequencing and bioinformatic methods expand at a rapid rate, so does the range of questions and predictions we can pose about gene expression, cell type identity, and their associations. Assumptions on what gene ought to be found in what cell type are pervasive throughout biology. While they are not all problematic, it is essential to acknowledge unconventional cases that could point us in the direction of novel relationships between gene expression and cell type identity. Here, we highlight such a case, where opsins are expressed in non-neuronal and progenitor cell types, which are not generally associated with light sensation, show the potential for high transcriptional activity, and share enriched TF binding motifs with genes associated with developmental patterning. While counter to canonical expectations for the opsin gene family, our findings can set a foundation on which to expand the role opsins play in the sensory and developmental processes in *Hydra*, and other metazoans.

## Author contributions

M.I.S.: study design, data curation, and analysis (substantial input from N.P., A.M.M., and T.H.O.), manuscript writing (substantial input from N.P., A.M.M., and T.H.O.), N.P.: study design, data analysis, and manuscript editing, A.M.M.: study design and manuscript editing, T.H.O.: study design, manuscript editing, and supervision.

## Supplementary Material

icaf100_Supplemental_Files

## Data Availability

A github repository for the analyses performed in this publication can be found at: https://github.com/mistoilova4/Hydra_scRNA_opsins The data underlying this article are available in the Dryad Digital Repository associated with Seibert et. al. (2019) at https://doi.org/10.5061/dryad.v5r6077 and the Hydra Genome Portal 2.0 at https://research.nhgri.nih.gov/hydra/. The URD Hydra Plotting Tutorial is available at https://github.com/cejuliano/hydra_single_cell/blob/master/URD_Hydra_Plotting_Tutorial.html.

## References

[bib1] Aguillon R, Rinsky M, Simon-Blecher N, Doniger T, Appelbaum L, Levy O. 2024. CLOCK evolved in cnidaria to synchronize internal rhythms with diel environmental cues.eLife. 12: RP89499. 38743049 10.7554/eLife.89499 PMC11093582

[bib2] Arshavsky VY, Burns ME. 2014. Current understanding of signal amplification in phototransduction. Madison (WI): Cellular Logistics. 10.4161/cl.29390 PMC4160332 25279249

[bib3] Arshavsky VY, Lamb TD, Jr PEN. 2002. G Proteins and phototransduction. Annu Rev Physiol. 64:153–87. 11826267 10.1146/annurev.physiol.64.082701.102229

[bib4] Asano M, Emori Y, Saigo K, Shiokawa K. 1992. Isolation and characterization of a *Xenopus* cDNA which encodes a homeodomain highly homologous to *Drosophila* distal-less. J Biol Chem. 267:5044–7. 1347527

[bib5] Berger CA, Tarrant AM. 2023. Sensory conflict disrupts circadian rhythms in the sea anemone. eLife. 12:e81084. 37022138 10.7554/eLife.81084 PMC10188108

[bib6] Bode HR . 2003. Head regeneration in *Hydra*. Dev Dyn. 226:225–36. 12557201 10.1002/dvdy.10225

[bib7] Bosch TCG, Anton-Erxleben F, Hemmrich G, Khalturin K. 2010. The *Hydra* polyp: nothing but an active stem cell community. Dev Growth Differ. 52:15–25. 19891641 10.1111/j.1440-169X.2009.01143.x

[bib8] Broun M, Bode HR. 2002. Characterization of the head organizer in hydra. Development. 129:875–84. 11861471 10.1242/dev.129.4.875

[bib9] Campbell RD, Marcum BA. 1980. Nematocyte migration in hydra: evidence for contact guidance in vivo. J Cell Sci. 41:33–51. 7364887 10.1242/jcs.41.1.33

[bib10] Castellano-Pellicena I, Uzunbajakava NE, Mignon C, Raafs B, Botchkarev VA, Thornton MJ. 2019. Does blue light restore human epidermal barrier function via activation of Opsin during cutaneous wound healing?. Lasers Surg Med. 51:370–82. 30168605 10.1002/lsm.23015

[bib11] Cazet JF, Siebert S, Little HM, Bertemes P, Primack AS, Ladurner P, Achrainer M, Fredriksen MT, Moreland RT, Singh S et al. 2023. A chromosome-scale epigenetic map of the genome reveals conserved regulators of cell state. Genome Res. 33:283–98. 36639202 10.1101/gr.277040.122 PMC10069465

[bib12] Chapman JA, Kirkness EF, Simakov O, Hampson SE, Mitros T, Weinmaier T, Rattei T, Balasubramanian PG, Borman J, Busam D et al. 2010. The dynamic genome of *Hydra*. Nature. 464:592–6. 20228792 10.1038/nature08830 PMC4479502

[doi54_386_032625] De Vivo Giacinto, Crocetta Fabio, Ferretti Miriam, Feuda Roberto, D’Aniello Salvatore, Battistuzzi Fabia Ursula. 2023. Duplication and Losses of Opsin Genes in Lophotrochozoan Evolution. Molecular Biology and Evolution, 40. 10.1093/molbev/msad066 PMC10097855 36947081

[bib13] Dupre C, Yuste R. 2017. Non-overlapping Neural Networks in *Hydra vulgaris*. Curr Biol. 27:1085–97. 28366745 10.1016/j.cub.2017.02.049 PMC5423359

[bib14] Farrell JA, Wang Y, Riesenfeld SJ, Shekhar K, Regev A, Schier AF. 2018. Single-cell reconstruction of developmental trajectories during zebrafish embryogenesis. Science. 360:6392. 10.1126/science.aar3131 PMC6247916 29700225

[bib15] Feuda R, Hamilton SC, McInerney JO, Pisani D. 2012. Metazoan opsin evolution reveals a simple route to animal vision. Proc Natl Acad Sci USA. 109:18868–72. 23112152 10.1073/pnas.1204609109 PMC3503164

[bib16] Feuda R, Menon AK, Göpfert MC. 2022. Rethinking Opsins. Mol Biol Evol. 39:msac033. 35143663 10.1093/molbev/msac033 PMC8892948

[bib17] Frazer SA, Baghbanzadeh M, Rahnavard A, Crandall KA, Oakley TH. 2024. Discovering genotype-phenotype relationships with machine learning and the Visual Physiology Opsin Database (VPOD). Gigascience. 13:giae073. 39460934 10.1093/gigascience/giae073 PMC11512451

[bib18] Gilbert E, Teeling C, Lebedeva T, Pedersen S, Chrismas N, Genikhovich G, Modepalli V. 2022. Molecular and cellular architecture of the larval sensory organ in the cnidarian *Nematostella vectensis*. Development. 149:dev200833. 36000354 10.1242/dev.200833 PMC9481973

[doi53_734_030625] Gornik Sebastian G, Bergheim Bruno Gideon, Morel Benoit, Stamatakis Alexandros, Foulkes Nicholas S, Guse Annika, Chang Belinda. 2021. Photoreceptor Diversification Accompanies the Evolution of Anthozoa. Molecular Biology and Evolution, 38:1744–1760. 10.1093/molbev/msaa304 33226083 PMC8097283

[bib20] Guertin S, Kass-Simon G. 2015. Extraocular spectral photosensitivity in the tentacles of *Hydra vulgaris*. Comp Biochem Physiol A Mol Integr Physiol. 184:163–70. 25724097 10.1016/j.cbpa.2015.02.016

[bib21] Hao W, Fong HK. 1999. The endogenous chromophore of retinal G protein-coupled receptor opsin from the pigment epithelium. J Biol Chem. 274:6085–90. 10037690 10.1074/jbc.274.10.6085

[bib22] Hunt DM, Hankins MW, Collin SP, Justin Marshall N. 2014. Evolution of visual and non-visual pigments. New York (NY): Springer.

[bib23] Hydra 2.0 Web Portal. 2025. (https://research.nhgri.nih.gov/hydra/. 11 June 2025, date last accessed).

[doi55_164_033025] Kanaya Hiroyuki J., Kobayakawa Yoshitaka, Itoh Taichi Q. 2019. Hydra vulgaris exhibits day-night variation in behavior and gene expression levels. Zoological Letters, 5. 10.1186/s40851-019-0127-1 PMC6407280 30891311

[bib24] Khan A, Fornes O, Stigliani A, Gheorghe M, Castro-Mondragon JA, van der Lee R, Bessy A, Chèneby J, Kulkarni SR, Tan G et al. 2018. JASPAR 2018: update of the open-access database of transcription factor binding profiles and its web framework. Nucleic Acids Res. 46:D1284. 29161433 10.1093/nar/gkx1188 PMC5753202

[bib25] Kingston ACN, Kuzirian AM, Hanlon RT, Cronin TW. 2015. Visual phototransduction components in cephalopod chromatophores suggest dermal photoreception. J Exp Biol. 218:1596–602. 25994635 10.1242/jeb.117945

[bib26] Lee AP, Koh EGL, Tay A, Brenner S, Venkatesh B. 2006. Highly conserved syntenic blocks at the vertebrate Hox loci and conserved regulatory elements within and outside Hox gene clusters. Proc Natl Acad Sci USA. 103:6994–9. 16636282 10.1073/pnas.0601492103 PMC1459007

[bib27] Leung NY, Montell C. 2017. Unconventional roles of Opsins. Annu Rev Cell Dev Biol. 33:241–64. 28598695 10.1146/annurev-cellbio-100616-060432 PMC5963513

[bib28] Liegertová M, Pergner J, Kozmiková I, Fabian P, Pombinho AR, Strnad H, Pačes J, Vlček Č, Bartůněk P, Kozmik Z. 2015. Cubozoan genome illuminates functional diversification of opsins and photoreceptor evolution. Sci Rep. 5:11885. 26154478 10.1038/srep11885 PMC5155618

[bib29] Lilly E, Muscala M, Sharkey CR, McCulloch KJ. 2024. Larval swimming in the sea anemone is sensitive to a broad light spectrum and exhibits a wavelength-dependent behavioral switch. Ecol Evol. 14:e11222. 38628921 10.1002/ece3.11222 PMC11019245

[bib30] Macias-Muñoz A, Murad R, Mortazavi A. 2019. Molecular evolution and expression of opsin genes in *Hydra vulgaris*. BMC Genomics. 20:992. 31847811 10.1186/s12864-019-6349-y PMC6918707

[bib31] Macosko EZ, Basu A, Satija R, Nemesh J, Shekhar K, Goldman M, Tirosh I, Bialas AR, Kamitaki N, Martersteck EM et al. 2015. Highly parallel genome-wide expression profiling of individual cells using nanoliter droplets. Cell. 161:1202–14. 26000488 10.1016/j.cell.2015.05.002 PMC4481139

[bib32] Marks PS . 1976. Nervous control of light responses in the sea anemone, *Calamactis praelongus*. J Exp Biol. 65:85–96. 11269 10.1242/jeb.65.1.85

[bib33] Murad R, Macias-Muñoz A, Wong A, Ma X, Mortazavi A. 2021. Coordinated gene expression and chromatin regulation during *Hydra* head regeneration. Genome Biol Evol. 13:evab221. 34877597 10.1093/gbe/evab221 PMC8651858

[bib34] Nordström K, Wallén R, Seymour J, Nilsson D. 2003. A simple visual system without neurons in jellyfish larvae. Proc R Soc Lond B. 270:2349–54. 10.1098/rspb.2003.2504 PMC1691513 14667350

[bib35] Picciani N, Kerlin JR, Sierra N, Swafford AJM, Ramirez MD, Roberts NG, Cannon JT, Daly M, Oakley TH. 2018. Prolific origination of eyes in cnidaria with co-option of non-visual Opsins. Curr Biol. 28:2413–2419.e4. 30033336 10.1016/j.cub.2018.05.055

[bib36] Plachetzki DC, Degnan BM, Oakley TH. 2007. The origins of novel protein interactions during animal opsin evolution. PLoS One. 2:e1054. 17940617 10.1371/journal.pone.0001054 PMC2013938

[bib37] Plachetzki DC, Fong CR, Oakley TH. 2012. Cnidocyte discharge is regulated by light and opsin-mediated phototransduction. BMC Biol. 10:17. 22390726 10.1186/1741-7007-10-17 PMC3329406

[bib38] Quiroga Artigas G, Lapébie P, Leclère L, Takeda N, Deguchi R, Jékely G, Momose T, Houliston E. 2018. A gonad-expressed opsin mediates light-induced spawning in the jellyfish *Clytia*. eLife. 7: e29555. 29303477 10.7554/eLife.29555 PMC5756024

[bib39] Rajamani R, Lin Y-L, Gao J. 2011. The opsin shift and mechanism of spectral tuning in rhodopsin. J Comput Chem. 32:854–65. 20941732 10.1002/jcc.21663 PMC3021771

[bib40] Ramirez MD, Pairett AN, Pankey MS, Serb JM, Speiser DI, Swafford AJ, Oakley TH. 2016. The last common ancestor of most bilaterian animals possessed at least 9 opsins. Genome Biol Evol. 8:3640–52. 28172965 10.1093/gbe/evw248 PMC5521729

[bib41] Ramirez MD, Speiser DI, Pankey MS, Oakley TH. 2011. Understanding the dermal light sense in the context of integrative photoreceptor cell biology. Vis Neurosci. 28:265–79. 21736861 10.1017/S0952523811000150

[bib42] Reddy PC, Gungi A, Ubhe S, Galande S. 2020. Epigenomic landscape of enhancer elements during *Hydra* head organizer formation. Epigenet Chromatin. 13:43. 10.1186/s13072-020-00364-6 PMC7552563 33046126

[bib43] Reid JE, Wernisch L. 2016. Pseudotime estimation: deconfounding single cell time series. Bioinformatics. 32:2973. 27318198 10.1093/bioinformatics/btw372 PMC5039927

[bib44] Schweikert LE, Bagge LE, Naughton LF, Bolin JR, Wheeler BR, Grace MS, Bracken-Grissom HD, Johnsen S. 2023. Dynamic light filtering over dermal opsin as a sensory feedback system in fish color change. Nat Commun. 14:4642. 37607908 10.1038/s41467-023-40166-4 PMC10444757

[bib45] Siebert S, Farrell JA, Cazet JF, Abeykoon Y, Primack AS, Schnitzler CE, Juliano CE. 2019. Stem cell differentiation trajectories in resolved at single-cell resolution. Science. 365:eaav9314. 31346039 10.1126/science.aav9314 PMC7104783

[bib46] Simeone A, Acampora D, Mallamaci A, Stornaiuolo A, D’Apice MR, Nigro V, Boncinelli E. 1993. A vertebrate gene related to orthodenticle contains a homeodomain of the bicoid class and demarcates anterior neuroectoderm in the gastrulating mouse embryo. EMBO J. 12:2735–47. 8101484 10.1002/j.1460-2075.1993.tb05935.x PMC413524

[bib47] Sinigaglia C, Busengdal H, Leclère L, Technau U, Rentzsch F. 2013. The bilaterian head patterning gene six3/6 controls aboral domain development in a cnidarian. PLoS Biol. 11:e1001488. 23483856 10.1371/journal.pbio.1001488 PMC3586664

[bib48] Speiser DI, Pankey MS, Zaharoff AK, Battelle BA, Bracken-Grissom HD, Breinholt JW, Bybee SM, Cronin TW, Garm A, Lindgren AR et al. 2014. Using phylogenetically-informed annotation (PIA) to search for light-interacting genes in transcriptomes from non-model organisms. BMC Bioinf. 15:350. 10.1186/s12859-014-0350-x PMC4255452 25407802

[bib49] Suda Y, Nakabayashi J, Matsuo I, Aizawa S. 1999. Functional equivalency between Otx2 and Otx1 in development of the rostral head. Development. 126:743–57. 9895322 10.1242/dev.126.4.743

[bib50] Suga H, Schmid V, Gehring WJ. 2008. Evolution and functional diversity of jellyfish opsins. Curr Biol. 18:51–5. 18160295 10.1016/j.cub.2007.11.059

[bib51] Technau U, Steele RE. 2011. Evolutionary crossroads in developmental biology: cnidaria. Development. 138:1447–58. 21389047 10.1242/dev.048959 PMC3062418

[bib52] Yokoyama S, Yang H, Starmer WT. 2008. Molecular basis of spectral tuning in the red- and green-sensitive (M/LWS) pigments in vertebrates. Genetics. 179:2037–43. 18660543 10.1534/genetics.108.090449 PMC2516078

