# Supplementary material for: Shedding Light on Patterns of Unconventional Expression of Opsin Genes in Hydra vulgaris
Source: Integr Comp Biol. 2025 Jun 23;65(3):648–60. doi: 10.1093/icb/icaf100 (PMC12464818; doi:10.1093/icb/icaf100)
Supplement: icaf100_Supplemental_Files [file icaf100_supplemental_files.zip › icb-2025-0124-File009.pdf]

Shedding light on unconventional opsin gene expression patterns in *Hydra vulgaris*

Table of Contents:

- Getting Started
  - Loading Packages and Data
- Plotting Opsin Expression
  - **Figure 2** : Plotting expression across cell types in *Hydra*
    - **Figure 2** 0.1% expression cutoff example
    - **Figure 2** 10% expression cutoff example
  - **Figure 3**: Plotting expression across cell states in *Hydra*
  - **Figure 4**: Plotting expression body sections in *Hydra*
  - misc. gene expression tests in discussion
    - Phototransduction cascade
    - Wnt
- Pulling Average Expression Values
- Pulling Metagene Scores

Getting Started

Loading Packages and Data

- I will warn that URD usually needs Seurat 2.3 not 5.2.1 and it was very difficult to get both to play nicely when generating the conda environment, there will be a yaml file in the repo but you may need to download some packages individually (see below)

- See Seurat site for installation: https://satijalab.org/seurat/articles/install.html
  source("https://z.umn.edu/archived-seurat")

- Install URD using these instructions:
  https://github.com/farrellja/URD/blob/0fed8b824644288ba4ec405ddf82a36960e2d4f9/INSTALL.md
  \

- I needed to download some dependencies individually before downloading URD
  conda install -c conda-forge --solver=libmamba r-nloptr
  conda install -c conda-forge --solver=libmamba r-lme4
  conda install -c conda-forge --solver=libmamba r-pbkrtest
  conda install -c conda-forge --solver=libmamba r-car
  conda install -c conda-forge --solver=libmamba r-vim

- I used this command to download URD
  Rscript -e 'source("https://raw.githubusercontent.com/farrellja/URD/master/URD-Install.R")'

In [1]:

```
#load packages
library("ggplot2")
library("Matrix")
library("URD")
library ("Seurat")
library("repr")
```

```
Registered S3 method overwritten by 'gplots':
  method         from
  reorder.factor gdata

Loading required package: SeuratObject

Loading required package: sp


Attaching package: ‘SeuratObject’


The following objects are masked from ‘package:base’:

    intersect, t
```

In [2]:

```
# CHANGE THIS TO REFLECT THE PATH WHERE YOU SAVED THE HYDRA DATA!
setwd("/kuhpc/scratch/cartwright/m026s156/hydrops/seibert_data")

# Define the output directory
output_dir <- "./output"

# Create it if it doesn't exist
if (!dir.exists(output_dir)) {
  dir.create(output_dir, recursive = TRUE)
}

# Load Hydra Transcriptome Object
hydra <- readRDS("URD_analysis_objects/Hydra_Seurat_Whole_Transcriptome.rds")

# Load URD objects with trees (i.e. branching trajectories)
interstitial <- readRDS("URD_analysis_objects/Hydra_URD_IC.rds")
ectoderm <- readRDS("URD_analysis_objects/Hydra_URD_Ectoderm.rds")
endoderm <- readRDS("URD_analysis_objects/Hydra_URD_Endoderm.rds")

# Load URD objects without trees (i.e. linear trajectories)
male <- readRDS("URD_analysis_objects/Hydra_URD_MaleTranscriptome.rds")
granular.zymogen <- readRDS("URD_analysis_objects/Hydra_URD_GranularZymogen.rds")
spumous <- readRDS("URD_analysis_objects/Hydra_URD_SpumousMucous.rds")

# Load spline curve fits for visualizing gene expression
ectoderm.splines <- readRDS("URD_analysis_objects/Splines-Ectoderm.rds")
endoderm.splines <- readRDS("URD_analysis_objects/Splines-Endoderm.rds")
male.splines <- readRDS("URD_analysis_objects/Splines-MaleTranscriptome.rds")
granular.zymogen.splines <- readRDS("URD_analysis_objects/Splines-GranularZymogen.rds")
spumous.splines <- readRDS("URD_analysis_objects/Splines-SpumousMucous.rds")

#Loading metagene data
meta <- load("gene_regulation_data/Candidate_Regulators/Enrichment_Results/corResults.Rd
id_inventory <- read.delim("op3_id_inventory.txt")
```

Plotting Opsin Expression

- This was adapted from the Plotting Hydra in URD tutorial from the Juliano Lab github: https://github.com/cejuliano/hydra_single_cell/blob/9d2dcb78155a616b22ae019b1c705d56738c1d38/[illegible]

find gene ID from URD objects

- pulled transcript codes from hydra dataset using PIA
- *Note:'t31971aep' seems to not match any gene in the interstitial annotation*

In [3]:
```
grep("t20729aep", rownames(interstitial@logupx.data), value=T)
grep("t20043aep", rownames(interstitial@logupx.data), value=T)
grep("t20044aep", rownames(interstitial@logupx.data), value=T)
grep("t29150aep", rownames(interstitial@logupx.data), value=T)
grep("t25412aep", rownames(interstitial@logupx.data), value=T)
grep("t3168aep", rownames(interstitial@logupx.data), value=T)
grep("t3233aep", rownames(interstitial@logupx.data), value=T)
grep("t32881aep", rownames(interstitial@logupx.data), value=T)
grep("t3169aep", rownames(interstitial@logupx.data), value=T)
grep("t21413aep", rownames(interstitial@logupx.data), value=T)
grep("t24564aep", rownames(interstitial@logupx.data), value=T)
grep("t31971aep", rownames(interstitial@logupx.data), value=T)
grep("t355aep", rownames(interstitial@logupx.data), value=T)
grep("t10575aep", rownames(interstitial@logupx.data), value=T)
grep("t24044aep", rownames(interstitial@logupx.data), value=T)
grep("t26793aep", rownames(interstitial@logupx.data), value=T)
grep("t9221aep", rownames(interstitial@logupx.data), value=T)
grep("t4885aep", rownames(interstitial@logupx.data), value=T)
grep("t15588aep", rownames(interstitial@logupx.data), value=T)
grep("t24989aep", rownames(interstitial@logupx.data), value=T)
grep("t27882aep", rownames(interstitial@logupx.data), value=T)
grep("t33805aep", rownames(interstitial@logupx.data), value=T)
grep("t37969aep", rownames(interstitial@logupx.data), value=T)
grep("t3337aep", rownames(interstitial@logupx.data), value=T)
grep("t36346aep", rownames(interstitial@logupx.data), value=T)
grep("t33516aep", rownames(interstitial@logupx.data), value=T)
grep("t33517aep", rownames(interstitial@logupx.data), value=T)
grep("t4602aep", rownames(interstitial@logupx.data), value=T)
grep("t26465aep", rownames(interstitial@logupx.data), value=T)
grep("t26466aep", rownames(interstitial@logupx.data), value=T)
grep("t29512aep", rownames(interstitial@logupx.data), value=T)
grep("t33568aep", rownames(interstitial@logupx.data), value=T)
grep("t14044aep", rownames(interstitial@logupx.data), value=T)
grep("t29959aep", rownames(interstitial@logupx.data), value=T)
grep("t17353aep", rownames(interstitial@logupx.data), value=T)
grep("t2106aep", rownames(interstitial@logupx.data), value=T)
grep("t21092aep", rownames(interstitial@logupx.data), value=T)
grep("t27688aep", rownames(interstitial@logupx.data), value=T)
grep("t36136aep", rownames(interstitial@logupx.data), value=T)
grep("t36280aep", rownames(interstitial@logupx.data), value=T)
grep("t4128aep", rownames(interstitial@logupx.data), value=T)
grep("t32850aep", rownames(interstitial@logupx.data), value=T)
grep("t31375aep", rownames(interstitial@logupx.data), value=T)
grep("t20210aep", rownames(interstitial@logupx.data), value=T)
grep("t16278aep", rownames(interstitial@logupx.data), value=T)
```

't20729aep|OPSP_COLLI'

't20043aep|OPSX_HUMAN'
't20044aep'
't29150aep|OPSX_HUMAN'
't25412aep|OPSO_SALSA'
't3168aep|OPN3_HUMAN'
't3233aep'
't32881aep|OPN3_MOUSE'
't3169aep|OPSX_HUMAN'
't21413aep|OPN4_PHOSU'
't24564aep|OPSR_CAPHI'
't31971aep'
't355aep|OPSG3_DANRE'
't10575aep|OPSR_BOVIN'
't24044aep|OPSP_ICTPU'
't26793aep|OPSO_RUTRU'
't9221aep|OPN3_HUMAN'
't4885aep|OPN5_MOUSE'
't15588aep|OPN3_HUMAN'
't27882aep|OPN3_HUMAN'
't33805aep|OPN4B_XENLA'
't37969aep|OPSD_ALLSU'
't3337aep'
't36346aep|OPN3_MOUSE'
't33516aep|OPSD2_MIZYE'
't33517aep|OPSD2_MIZYE'
't4602aep'
't26465aep|OPN3_HUMAN'
't26466aep|OPN3_HUMAN'
't29512aep|OPSP_CHICK'
't33568aep'
't14044aep|OPSD2_MIZYE'
't29959aep|OPN4B_XENLA'
't17353aep|OPN4B_XENLA'
't2106aep|OPN3_HUMAN'
't21092aep|OPSD_LOLFO'
't27688aep|OPSP_COLLI'
't36136aep|OPSD_LOLFO'
't36280aep|OPS2_DROPS'
't4128aep|OPSD2_MIZYE'
't32850aep'
't31375aep|OPSC1_HEMSA'
't20210aep'
't16278aep|OPSC2_HEMSA'

Figure 2 : Plotting expression across cell types in *Hydra*

In [4]:

```
# updates V2 seurat object to V3 (changes | and _ to - in names)
hydraV3 <- UpdateSeuratObject(hydra)

#get list of opsin
new.hydraV3.features <- c(
  "t20729aep-OPSP-COLLI", "t20043aep-OPSX-HUMAN", "t20044aep", "t29150aep-OPSX-HUMAN", "
  "t3168aep-OPN3-HUMAN", "t3233aep", "t32881aep-OPN3-MOUSE", "t3169aep-OPSX-HUMAN", "t21
  "t24564aep-OPSR-CAPHI", "t31971aep", "t355aep-OPSG3-DANRE", "t10575aep-OPSR-BOVIN", "t
  "t26793aep-OPSO-RUTRU", "t9221aep-OPN3-HUMAN", "t4885aep-OPN5-MOUSE", "t15588aep-OPN3-
  "t33805aep-OPN4B-XENLA", "t37969aep", "t3337aep", "t36346aep-OPN3-MOUSE", "t33516aep-O
  "t4602aep", "t26465aep-OPN3-HUMAN", "t26466aep-OPN3-HUMAN", "t29512aep-OPSP-CHICK", "t
  "t29959aep-OPN4B-XENLA", "t17353aep-OPN4B-XENLA", "t2106aep-OPN3-HUMAN", "t21092aep-OP
  "t36136aep-OPSD-LOLFO", "t36280aep-OPS2-DROPS", "t4128aep-OPSD2-MIZYE", "t31375aep-OPS
  "t24989aep", "t32850aep"
)

#To reorder cluster ID's for better readability
#Get the Orders:
#current_levels <- levels(hydraV3)
#print(current_levels)

#make new order
new_order <- c(
  "ecEp_stem_cell", "ecEp-nb(pd)", "ecEp-nem(id)", "ecEp_basal_disk", "ecEp_head",
  "ecEp_battery_cell1(mp)", "ecEp_battery_cell2(mp)", "enEp_stem_cell", "enEp-nb(pd)",
  "enEp-nem(pd)", "enEp_tent-nem(pd)", "enEp_foot", "enEp_head", "enEp_tentacle",
  "i_stem_cell/progenitor", "i_nematocyte", "i_nb1", "i_nb2", "i_nb3", "i_nb4", "i_nb5",
  "i_granular_mucous_gland_cell", "i_spumous_mucous_gland_cell", "i_zymogen_gland_cell",
  "i_male_germline", "i_female_germline1", "i_female_germline2_nurse",
  "i_neuron/gland_cell_progenitor", "i_neuron_progenitor", "i_neuron_ec1", "i_neuron_ec2
  "i_neuron_ec3", "i_neuron_ec4", "i_neuron_ec5", "i_neuron_en1", "i_neuron_en2",
  "i_neuron_en3", "db")

#reassign new order to hydra V3
levels(hydraV3) <- new_order
```

```
Updating from v2.X to v3.X

Warning message:
“Not validating Assay objects”
Warning message:
“Not validating Assay objects”
Warning message:
“Not validating DimReduc objects”
Warning message:
“Not validating DimReduc objects”
Warning message:
“Not validating DimReduc objects”
Warning message:
“Not validating DimReduc objects”
Warning message:
“Not validating Seurat objects”
Warning message:
“Not validating Seurat objects”
Warning message:
“Not validating Seurat objects”
Validating object structure

Updating object slots

Ensuring keys are in the proper structure

Updating matrix keys for DimReduc ‘pca’

Updating matrix keys for DimReduc ‘tsne’

Ensuring keys are in the proper structure

Ensuring feature names don't have underscores or pipes

Warning message:
“Not all features provided are in this Assay object, removing the following feature(s): t
34446aep|VIAAT-XENLA, t35904aep|DDAC-ENTAG, t31833aep|BP10-PARLI, t38117aep|SAXO1-RAT, t3
5860aep|KIF3A-MACFA, t35863aep|SSPO-CHICK, t32307aep|NCAN-PANTR, t35991aep|FLNC-RAT, t318
44aep|TLL1-DANRE, t31227aep|SYCP2-RAT, t37814aep|CC105-BOVIN, t32265aep|SYD1-CAEEL, t3226
6aep|GGT1-PIG, t32263aep|IF172-DANRE, t33696aep|CNRP1-BOVIN, t35853aep|TM175-MOUSE, t3585
0aep|CYTA-MOUSE, t37320aep|CO6A6-HUMAN, t32616aep|FBN1-BOVIN, t32412aep|M3K19-HUMAN, t324
13aep|NTPES-BACSU, t36940aep|ROP1L-XENLA, t36938aep|SYT15-HUMAN, t31713aep|TBA-NOTVI, t37
623aep|RHES-HUMAN, t36574aep|OPN4B-XENLA, t36585aep|CALM-MACPY, t38683aep|H10A-XENLA, t38
674aep|S7A14-HUMAN, t32852aep|NPC2-DROME, t36486aep|SCN1-HETBL, t34600aep|KPSH1-BOVIN, t3
2785aep|HEBP1-MOUSE, t36257aep|EDIL3-MOUSE, t38455aep|PDE11-DROME, t31915aep|F149A-MOUSE,
t34560aep|AMPN-RAT, t35296aep|MORN2-BOVIN, t31908aep|RSLBA-HUMAN, t31899aep|AT1A-HYDVU, t
34195aep|SAM9L-HUMAN, t34183aep|TLN1-HUMAN, t32775aep|CELR2-HUMAN, t33155aep|CBPD-LOPSP,
t36928aep|CACB2-RABIT, t35006aep|CER1-HUMAN, t36031aep|ANPRA-MOUSE, t34090aep|MYLK-RABIT,
t32136aep|ZDH11-MOUSE, t32151aep|HE-PARLI, t38447aep|SAX-LITCT, t34475aep|LRIG2-HUMAN, t3
7718aep|CFA45-HUMAN, t37723aep|INTU-BOVIN, t32514aep|NPHP1-CANLF, t36994aep|KINH-SYNRA, t
35151aep|ACTP1-ANTAS, t35152aep|ACTP1-ANTAS, t37988aep|GLNA-PANAR, t33783aep|GRM3-MOUSE,
t31950aep|B3GN2-HUMAN, t31935aep|AP4S1-HUMAN, t31942aep|ECE1-CAVPO, t32744aep|LMX1A-HUMA
N, t33595aep|FLT3-HUMAN, t38581aep|HE-HEMPU, t38586aep|KCNB1-PIG, t38428aep|SIX6-HUMAN, t
38432aep|MYO3B-HUMAN, t38498aep|MARH1-HUMAN, t33119aep|KINH-STRPU, t36612aep|ERIC3-HUMAN,
t32286aep|RIMS2-RAT, t32909aep|F228B-MOUSE, t32328aep|SPIT3-HUMAN, t32896aep|LOX5-RAT, t3
7679aep|DCC-MOUSE, t33218aep|TACC3-XENLA, t33224aep|CD047-MOUSE, t33213aep|CEP44-XENTR, t
33869aep|OSBL8-MOUSE, t37402aep|NAC1-CAVPO, t37405aep|OTOF-DANRE, t32202aep|B4GN4-HUMAN,
t32189aep|NINL-HUMAN, t38735aep|KAPR2-DROME, t38172aep|CCD13-HUMAN, t37761aep|ADRB2-MOUS
E, t31410aep|ANKF1-HUMAN, t31430aep|ARGI1-HUMAN, t37782aep|EMAL4-XENTR, t35785aep|KCNA1-R
AT, t34532aep|TFF3-RAT, t34497aep|CHIT3-DROME, t37145aep|ZCPW1-HUMAN, t37156aep|CHIA-MOUS
E, t31809aep|KPCA-RAT, t31783aep|DMBT1-RABIT, t36106aep|USH1C-MOUSE, t31356aep|OTOF-HUMA
```

N, t31354aep|CA2D4-HUMAN, t31349aep|GRM7-HUMAN, t31357aep|LRRC9-HUMAN, t37266aep|HARB1-HU
MAN, t37247aep|NAS13-CAEEL, t37309aep|WHRN-HUMAN, t31480aep|KCNB2-MOUSE, t34425aep|ZMYM1-
HUMAN, t34424aep|TRY3-SALSA, t32564aep|AVIL-HUMAN, t32529aep|LOXH1-MOUSE, t32002aep|HMCN1
-HUMAN, t32011aep|SAL-SILAS, t35471aep|FHAD1-HUMAN, t35503aep|DSCL1-CHICK, t35576aep|ZBED
4-MOUSE, t34201aep|RTJK-DROME, t34267aep|H2B3-TIGCA, t34240aep|ZMYM1-HUMAN, t34261aep|AGR
L3-BOVIN, t37937aep|AL14E-RAT, t37855aep|THAP9-HUMAN, t37336aep|ZMYM1-HUMAN, t37327aep|TC
B2-CAEBR, t31767aep|FKBP9-RAT, t34857aep|U390-DANRE, t34853aep|FUT4-HUMAN, t34824aep|PCR3
-ARATH, t35637aep|RNF11-MOUSE, t38527aep|ANK1-HUMAN, t33129aep|LSM1-HUMAN, t32130aep|NPC2
-DROME, t36020aep|ADCY9-MOUSE, t36201aep|B4GN4-HUMAN, t33788aep|MCAF1-HUMAN, t34900aep|ML
C2-DROME, t36537aep|BOULE-DROME, t34831aep|MTH2-DROYA, t36301aep|MCPI-MELCP, t34733aep|MC
PI-MELCP, t36216aep|DLP1-MOUSE, t34780aep|Y1101-SYNY3, t33829aep|MLP-ACRMI, t36240aep|CDK
L1-DANRE, t36310aep|CPI1-PIG, t32653aep|DRAM2-MOUSE, t32647aep|DD3-DICDI, t33926aep|TEAD1
-HUMAN, t37841aep|PYRD1-BOVIN, t37085aep|STK16-RAT, t26085aep|TBA-LYTPI, t26076aep|JAZF1-
MOUSE, t20399aep|ELAV2-XENTR, t1163aep|NOT2-XENLA, t5706aep|YXIE-BACSU, t28847aep|CC191-H
UMAN, t28848aep|KAD5-BOVIN, t18735aep|FOXA2-ORYLA, t28510aep|CEP83-MOUSE, t28512aep|RAP1-
CAEEL, t28513aep|LAR-CAEEL, t4095aep|PNCA-ECOLI, t26432aep|KCNK9-XENLA, t7698aep|MFNA-MET
PE, t28473aep|GAPR1-HUMAN, t14437aep|FGF1-XENLA, t27792aep|MLP-ACRMI, t26387aep|GLAS-DROV
I, t26394aep|TMED7-HUMAN, t29717aep|RWA1-ARATH, t29715aep|CDK14-CALJA, t29720aep|AD26A-MO
USE, t29725aep|BRAC-CHICK, t3271aep|IGS10-HUMAN, t24865aep|AGRB3-MOUSE, t26158aep|VMO1-HU
MAN, t26163aep|GGH-MOUSE, t18765aep|EAA1-HUMAN, t25706aep|PRFA-POLPE, t25721aep|CA158-HUM
AN, t5096aep|WDR25-HUMAN, t21322aep|SOX14-DANRE, t7296aep|CO1A2-LITCT, t7297aep|CO4A2-ASC
SU, t4550aep|CNIF3-ARATH, t4557aep|CBPB-ASTAS, t4539aep|CEL2A-PIG, t4522aep|CALM-SCHPO, t
28181aep|LMOD3-DANRE, t13955aep|KCNB2-RABIT, t28188aep|AQP9-HUMAN, t1197aep|SP17-RABIT, t
18829aep|HPCL1-SHEEP, t23544aep|PRY1-YEAST, t23523aep|CALM-DICDI, t23543aep|DIRA1-HUMAN,
t10102aep|CETN1-BOVIN, t24351aep|FUT11-RAT, t11055aep|LWA-HYDEC, t11063aep|G3ST3-MOUSE, t
3676aep|TES-CHICK, t3666aep|PNCB-DROME, t14aep|MFSD6-PIG, t33aep|SCLY-BOVIN, t17610aep|S1
7B1-XENLA, t17596aep|ZRAN1-HUMAN, t17590aep|NCAH-DROME, t29189aep|ANPRA-MOUSE, t17607aep|
LRC73-HUMAN, t24795aep|P4HTM-HUMAN, t3206aep|PACN1-PONAB, t12108aep|STXB4-MOUSE, t27585ae
p|DRD1-PIG, t16624aep|FKB14-MOUSE, t13326aep|KLF13-HUMAN, t26415aep|ATS7-RAT, t28460aep|P
GPS1-CHICK, t19638aep|HEBP2-HUMAN, t29694aep|RLBP1-HUMAN, t25237aep|ASIC3-HUMAN, t3968aep
|NDF4-HUMAN, t25235aep|CHAC2-DANRE, t3974aep|ELAV2-XENTR, t7408aep|NALD2-MOUSE, t26356aep
|NR1BA-DANRE, t7418aep|TYRO-STRGA, t7419aep|MAF1-MOUSE, t12001aep|MORN4-BOVIN, t12013aep|
DYRK2-MOUSE, t1821aep|PP3BB-XENLA, t1806aep|HEXA-HUMAN, t3545aep|P4HTM-MOUSE, t3538aep|CP
EB1-PONAB, t25019aep|GA2L1-MOUSE, t25014aep|KLF5-MOUSE, t29421aep|PRY3-YEAST, t29417aep|O
GA-RAT, t12027aep|ECE1-CAVPO, t7359aep|FKBP7-PONAB, t26322aep|ZIFL1-ARATH, t7356aep|EGL44
-CAEEL, t1509aep|FOXI2-XENTR, t1499aep|NAS13-CAEEL, t25826aep|UGDH-MOUSE, t25835aep|FAT2-
DROME, t20372aep|TT30A-XENTR, t27323aep|CRYAB-BOVIN, t25458aep|DYH2-MOUSE, t29021aep|CC14
2-MOUSE, t29029aep|ABHD3-MOUSE, t30881aep|DAPK2-MOUSE, t27148aep|ADRB2-TSCTR, t27165aep|A
IDA-DANRE, t10418aep|QPCTL-BOVIN, t10412aep|TXD17-PONAB, t22547aep|ASIC1-HUMAN, t25400aep
|TRAF4-MOUSE, t18888aep|TFP8L-DROPS, t25389aep|PTPR2-HUMAN, t18857aep|PRY1-YEAST, t28780a
ep|MLP-ACRMI, t16101aep|EVA1C-HUMAN, t18862aep|SLIT-DROME, t16977aep|TBB-HALDI, t29009aep
|PHAR2-HUMAN, t29004aep|FGRL1-RAT, t31088aep|ZMY11-MOUSE, t28493aep|CH074-BOVIN, t28503ae
p|PPIB-CHICK, t24455aep|SEPR-MOUSE, t27833aep|CL056-HUMAN, t29581aep|BMPH-STRPU, t27525ae
p|MUC5B-CHICK, t27530aep|HYDMA-HYDVU, t25340aep|CC177-MOUSE, t25337aep|CALM-PNECA, t13247
aep|GGT1-HUMAN, t13256aep|CSL3-ONCKE, t26873aep|ZSC31-HUMAN, t26867aep|PDE11-MOUSE, t2579
0aep|B4GN3-MOUSE, t24660aep|TRIM9-HUMAN, t24667aep|CALM-PROMN, t18612aep|ACE-RAT, t18616a
ep|PRY1-YEAST, t9164aep|GRM8-MOUSE, t14360aep|TSEAR-MOUSE, t28440aep|CALM-HETTR, t28433ae
p|CAS4-EPHMU, t27539aep|LOXH1-MOUSE, t2568aep|GCY-STRPU, t2573aep|RGMA-MOUSE, t24874aep|E
CE1-MOUSE, t3297aep|MMP27-HUMAN, t9165aep|SEC4-CANGA, t15140aep|KCNF1-HUMAN, t28605aep|EN
TP1-HUMAN, t15178aep|MOT14-MOUSE, t15139aep|B3GA3-MOUSE, t26771aep|CATL-SARPE, t13791aep|
OSGI2-HUMAN, t13822aep|MEIG1-XENLA, t13811aep|TSP2-MOUSE, t2765aep|LIN32-CAEEL, t2754aep|
SYCE2-MOUSE, t24627aep|CAC1A-RAT, t24623aep|LOX5-MESAU, t2746aep|1433-DICDI, t29633aep|AN
K2-HUMAN, t30843aep|LPAR6-RAT, t30835aep|MCATL-BOVIN, t30836aep|GBRB3-RAT, t6334aep|ZNT10
-MOUSE, t17347aep|NAS4-CAEEL, t17340aep|SERC3-PONAB, t29082aep|KCNF1-HUMAN, t29240aep|NDK
7-MOUSE, t29244aep|YJBM-BACSU, t23435aep|DLX1A-DANRE, t31040aep|ACH10-HUMAN, t23446aep|SA
MH1-HUMAN, t27007aep|GFI1B-CHICK, t26993aep|FEZF2-BOVIN, t10197aep|COCA1-CHICK, t10214aep
|ENDB1-SOLTU, t10702aep|P4HTM-HUMAN, t10714aep|KSR2-MOUSE, t10711aep|CALM-SOLLC, t30059ae
p|LMIP-BOVIN, t25085aep|COCA1-CHICK, t3809aep|PRFA-POLPE, t18810aep|NAS4-CAEEL, t27685aep
|NCKX5-DANRE, t27673aep|CAPA-BACAN, t27676aep|MYO3A-HUMAN, t30358aep|RHO4-SCHPO, t30362ae
p|KCNC1-MOUSE, t26205aep|CHSTB-MOUSE, t30962aep|RDH13-MOUSE, t7790aep|YJBQ-ECOLI, t7785ae
p|PPR3B-DANRE, t26459aep|MAPK5-HUMAN, t26298aep|CO6A6-HUMAN, t24960aep|TRIM3-RAT, t3398ae
p|EVA1C-MOUSE, t24945aep|CNNM2-RAT, t3388aep|PDP2-RAT, t23376aep|COMA-CONMA, t23364aep|DY

```
I2-HELCR, t31010aep|KLH17-HUMAN, t31013aep|CSL3-ONCKE, t31006aep|P4HA1-CHICK, t31009aep|P
RDM6-MOUSE, t23352aep|GREM2-MOUSE, t30991aep|MSH2-CHLAE, t22700aep|NDF1-DANRE, t30802aep|
BLML4-DICDI, t25746aep|LY75-HUMAN, t25763aep|AGRIN-HUMAN, t25738aep|H2AL-STRPU, t25739aep
|H2AL-STRPU, t5157aep|ADCY9-HUMAN, t17394aep|CD151-BOVIN, t17378aep|LOXH1-HUMAN, t24828ae
p|E”
Updating slots in RNA

Updating slots in pca

Updating slots in tsne

Setting tsne DimReduc to global

Validating object structure for Assay ‘RNA’

Validating object structure for DimReduc ‘pca’

Validating object structure for DimReduc ‘tsne’

Object representation is consistent with the most current Seurat version
```

Figure 2 Plot

In [6]:
```
DotPlot(
        object = hydraV3,
        features = new.hydraV3.features,
        cols = c("lightgrey", "black"),
        dot.scale = 6 ,
        dot.min = 0.05, #setting percent expression threshold
        #scale.min =  ,
        cluster.idents = FALSE,
        scale = FALSE, # Make TRUE if you want log normalized, read corrected counts to
        scale.by = "size",
)+ RotatedAxis()
options(repr.plot.width=15, repr.plot.height=10)
```

```
Warning message:
“The following requested variables were not found: t37969aep, t24989aep”
Warning message:
“Removed 1591 rows containing missing values or values outside the scale range
(`geom_point()`).”
```

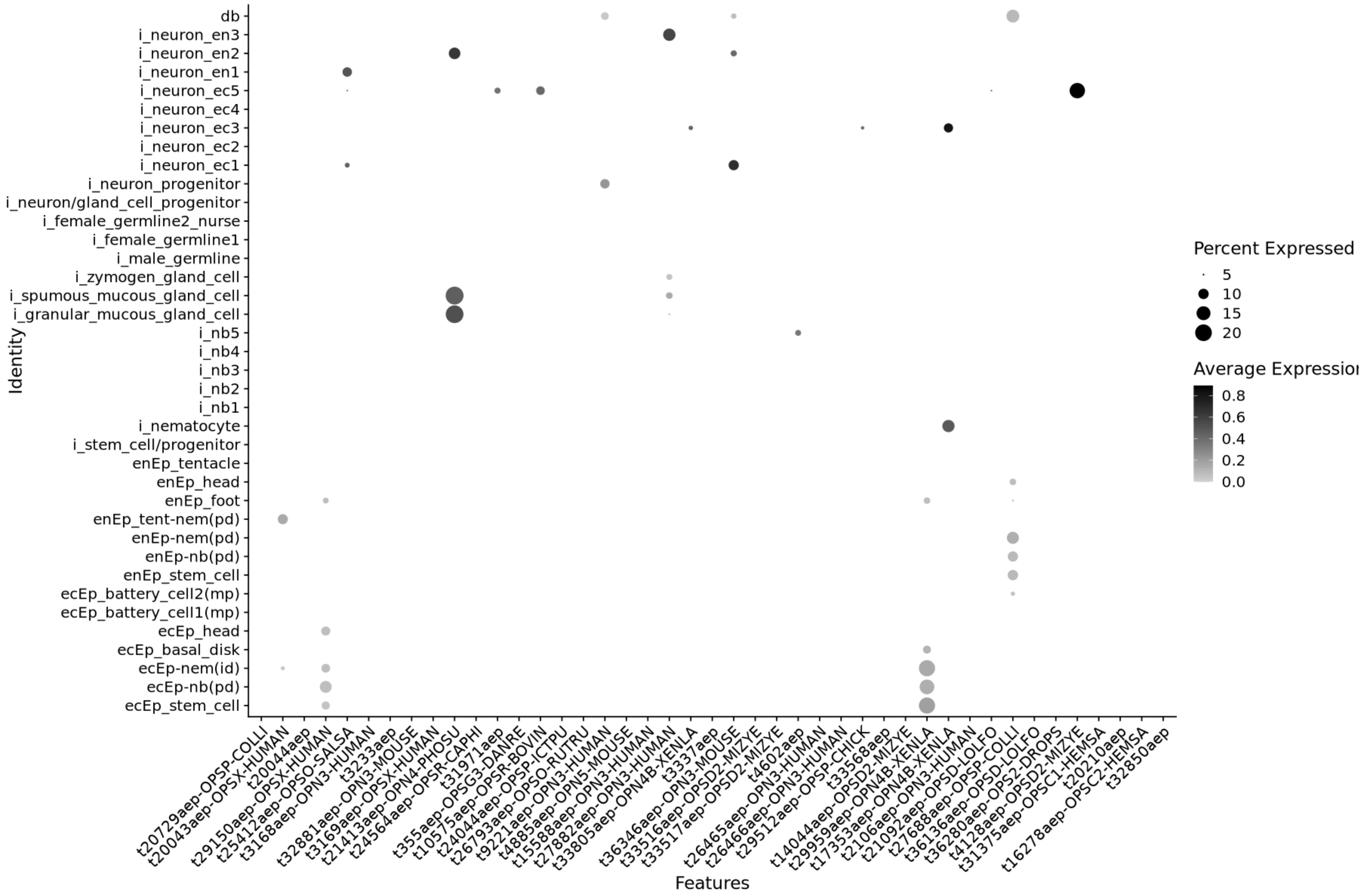

In [7]:
```
#If you want PDF output un comment the next and last line

output_file <- file.path(output_dir, "Figure_3_dotplot_5p.tif")
tiff(output_file, width = 15, height = 10, units = "in", res = 300)
DotPlot(
        object = hydraV3,
        features = new.hydraV3.features,
        cols = c("lightgrey", "black"),
        dot.scale = 6 ,
        dot.min = 0.05, #setting percent expression threshold
        #scale.min =  ,
        cluster.idents = FALSE,
        scale = FALSE, # Make TRUE if you want log normalized, read corrected counts to
        scale.by = "size",
)+ RotatedAxis()
dev.off()
#resize plot
#options(repr.plot.width=15, repr.plot.height=10)
```

```
Warning message:
“The following requested variables were not found: t37969aep, t24989aep”
Warning message:
“Removed 1591 rows containing missing values or values outside the scale range
(`geom_point()`).”
```

pdf: 2

Figure 2: 0.1% expression cutoff example

In [8]:
```
#making opsin dotplot
#If you want PDF output un comment the next and last line
#pdf("./output/Figure_2_dotplot_0.1p.pdf", width = 15, height = 10)
DotPlot(
```

```
        object = hydraV3,
        features = new.hydraV3.features,
        cols = c("lightgrey", "black"),
        dot.scale = 6 ,
        dot.min = 0.001, #setting percent expression threshold
        cluster.idents = FALSE,
        scale = FALSE,
        scale.by = "size",
)+ RotatedAxis()
#dev.off()
#resize plot
options(repr.plot.width=15, repr.plot.height=10)
```

```
Warning message:
“The following requested variables were not found: t37969aep, t24989aep”
Warning message:
“Removed 1014 rows containing missing values or values outside the scale range
(`geom_point()`).”
```

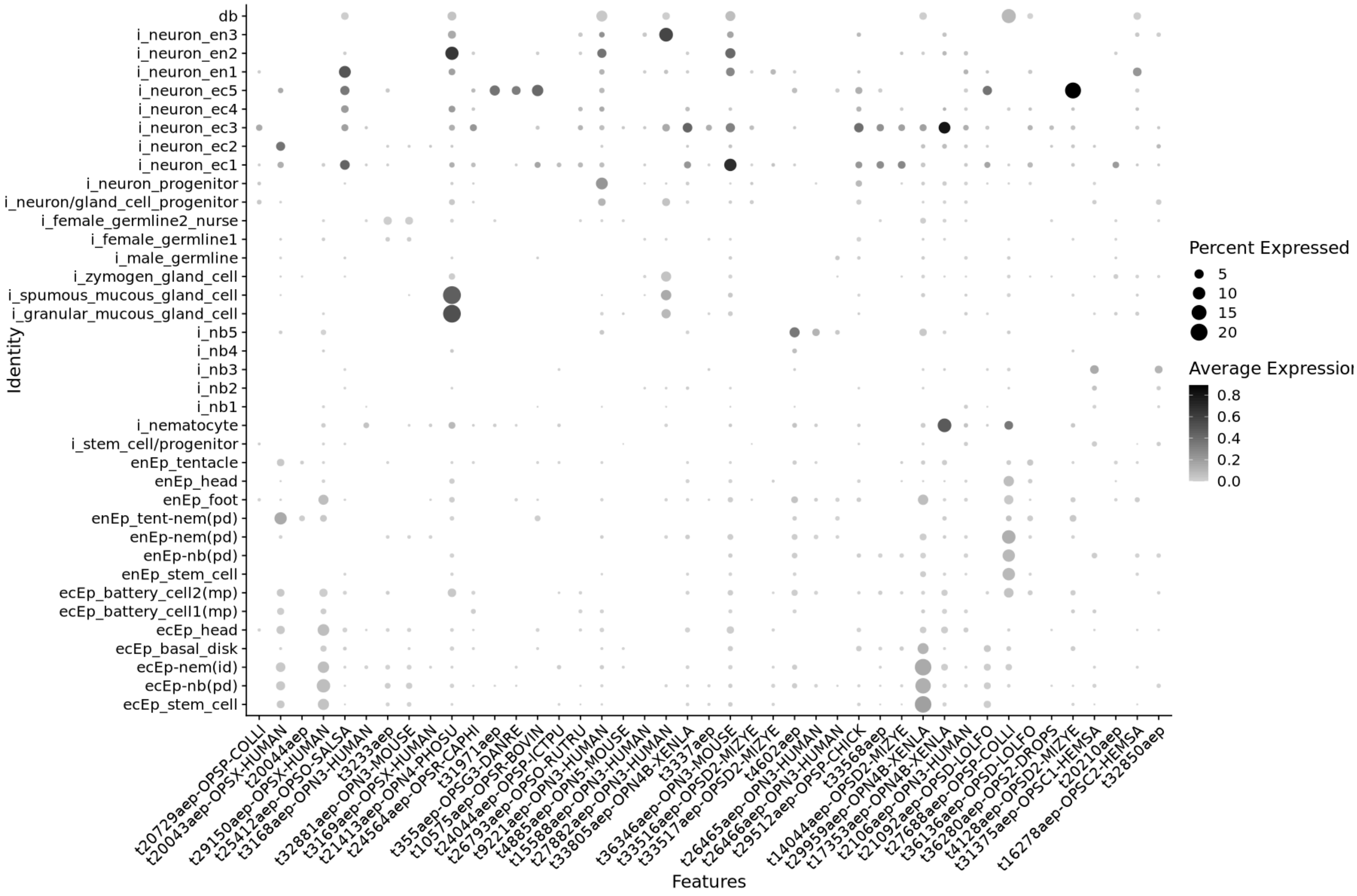

Figure 2: 10% expression cutoff example

In [10]:
```
#If you want PDF output un comment the next and last line
#pdf("./output/Figure_2_dotplot_10p.pdf", width = 15, height = 10)
DotPlot(
        object = hydraV3,
        features = new.hydraV3.features,
        cols = c("lightgrey", "black"),
        dot.scale = 6 ,
        dot.min = 0.1, #setting percent expression threshold
        cluster.idents = FALSE,
        scale = FALSE,
        scale.by = "size",
)+ RotatedAxis()
#dev.off()
```

```
#resize plot
options(repr.plot.width=15, repr.plot.height=10)
```

```
Warning message:
“The following requested variables were not found: t37969aep, t24989aep”
Warning message:
“Removed 1621 rows containing missing values or values outside the scale range
(`geom_point()`).”
```

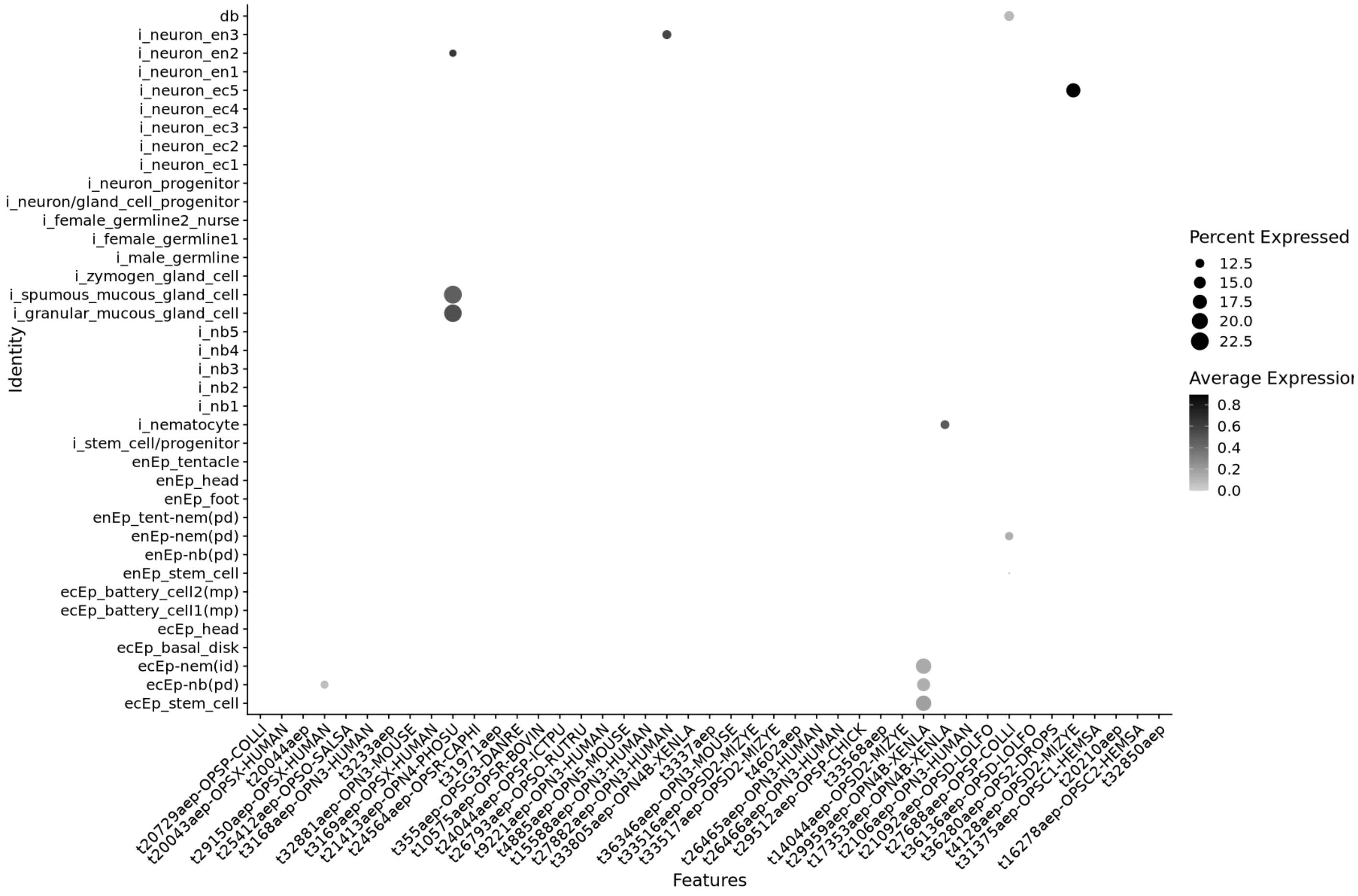

Figure 3: Plotting expression across cell states in *Hydra*

In [11]:
```
# Export opsin plot on interstitial cell lineage
output_file <- file.path(output_dir, "Figure_2_t21413aep_interstitial.tif")
tiff(output_file, width = 10, height = 10, units = "in", res = 300)
plotTree(interstitial,
        "t21413aep|OPN4_PHOSU",
         tree.size = 4,
         cell.size = 2,
         color.limits = c(0,3)
        )
dev.off()

# Export opsin plot on ectodermal cell lineage
output_file <- file.path(output_dir, "Figure_3_t29959aep_ectodermal.tif")
tiff(output_file, width = 10, height = 10, units = "in", res = 300)
plotTree(ectoderm,
         "t29959aep|OPN4B_XENLA",
         tree.size = 4,
         cell.size = 2,
         color.limits = c(0,3)
        )
dev.off()
```

```
# Export opsin plot on endodermal lineage
output_file <- file.path(output_dir, "Figure_3_t27688aep_endodermal.tif")
tiff(output_file, width = 10, height = 10, units = "in", res = 300)
plotTree(endoderm,
         "t27688aep|OPSP_COLLI",
         tree.size = 4,
         cell.size = 2,
         color.limits = c(0,3)
         )
dev.off()
```

pdf: 2

pdf: 2

pdf: 2

Figure 4 : Plotting expression body sections in *Hydra*

In [12]:

```
output_file <- file.path(output_dir, "Figure_4_dotplot_5.tif")
tiff(output_file, width = 15, height = 10, units = "in", res = 300)
plotSmoothFitMultiCascade(ectoderm.splines, c("t29150aep|OPSX_HUMAN",
                                              "t20043aep|OPSX_HUMAN",
                                              "t21092aep|OPSD_LOLFO",
                                              "t29959aep|OPN4B_XENLA"), ncol = 1)
dev.off()
```

pdf: 2

Other Gene Expression Patterns

Phototransduction cascade

In [13]:

```
# Phototransduction Cascade Genes from Macias-Munoz et al. 2019 table S3

#Gαs
grep("t12724aep", rownames(interstitial@logupx.data), value=T)
#Gαo
grep("t15699aep", rownames(interstitial@logupx.data), value=T)
#Gαi
grep("t12031aep", rownames(interstitial@logupx.data), value=T)
#Gɣ1
grep("t13138aep", rownames(interstitial@logupx.data), value=T)
#Gɣ2
grep("t26652aep", rownames(interstitial@logupx.data), value=T)
#GRK5-like
grep("t13671aep", rownames(interstitial@logupx.data), value=T)
#GMP-PDEα1
grep("t26867aep", rownames(interstitial@logupx.data), value=T)
#GMP-PDEα2
grep("t38455aep", rownames(interstitial@logupx.data), value=T)
#GMP-PDEβ
grep("t20025aep", rownames(interstitial@logupx.data), value=T)
#GMP-PDEΔ
grep("t25255aep", rownames(interstitial@logupx.data), value=T)
#Phosphodiesterase
grep("t6798aep", rownames(interstitial@logupx.data), value=T)
#CNG
grep("t27655aep", rownames(interstitial@logupx.data), value=T)
#RGS12-like
grep("t26213aep", rownames(interstitial@logupx.data), value=T)
```

```
#ANPR1-like
grep("t7063aep", rownames(interstitial@logupx.data), value=T)
#ANPR1-like2
grep("t21324aep", rownames(interstitial@logupx.data), value=T)
#Neurocalcin-like
grep("t38370aep", rownames(interstitial@logupx.data), value=T)
#Gαq
grep("t612aep", rownames(interstitial@logupx.data), value=T)
#PLC
grep("t29781aep", rownames(interstitial@logupx.data), value=T)
#Ankyrin-3-like
grep("t27547aep", rownames(interstitial@logupx.data), value=T)
#Gβ1
grep("t19432aep", rownames(interstitial@logupx.data), value=T)
#Rh kinase
grep("t17074aep", rownames(interstitial@logupx.data), value=T)
#Arrestin
grep("t14420aep", rownames(interstitial@logupx.data), value=T)
#SEC14-like
grep("t7389aep", rownames(interstitial@logupx.data), value=T)

#Additional sequences
#PREDICTED: Hydra vulgaris homeobox protein 2 (LOC124816966), mRNA
grep("t15272aep", rownames(interstitial@logupx.data), value=T)
#ANR28_HUMAN
grep("t911aep", rownames(interstitial@logupx.data), value=T)
#ANR28_MOUSE
grep("t27546aep", rownames(interstitial@logupx.data), value=T)
#ANK1_HUMAN
grep("t11518aep", rownames(interstitial@logupx.data), value=T)
grep("t32411aep", rownames(interstitial@logupx.data), value=T)
grep("t36031aep", rownames(interstitial@logupx.data), value=T)
grep("t28209aep", rownames(interstitial@logupx.data), value=T)
grep("t2619aep", rownames(interstitial@logupx.data), value=T)
grep("t30156aep", rownames(interstitial@logupx.data), value=T)
grep("t25553aep", rownames(interstitial@logupx.data), value=T)
```

't12724aep|GNAS_HOMAM'

't15699aep|GNAO_BOVIN'

't12031aep|GNAI_PATPE'

't13138aep|GBG2_PONAB'

't26652aep|GBG7_HUMAN'

't13671aep|GRK5_BOVIN'

't26867aep|PDE11_MOUSE'

't38455aep|PDE11_DROME'

't20025aep|PDE6_DROMO'

't25255aep|PDE6D_MOUSE'

't6798aep|PDE9A_PANTR'

't27655aep|CNGA3_HUMAN'

't26213aep|RGS12_RAT'

't7063aep|GCY_STRPU'

't21324aep|GC76C_DROME'

't38370aep|NCAH_DROME'

't612aep|GNAQ_MIZYE'

't29781aep|PIP1_DROME'

't27547aep|ANK3_RAT'

't19432aep|GBB_PINFU'

't17074aep|ARBK2_BOVIN'

't14420aep|ARRB1_MACFA'

't7389aep|S14L1_HUMAN'

't15272aep'

't911aep|ANR28_HUMAN'

't27546aep|ANR28_MOUSE'

't11518aep|ANK1_HUMAN'

't32411aep|CNGA2_BOVIN'

't36031aep|ANPRA_MOUSE'

't28209aep'

't2619aep|PIP1_DROME'

't30156aep|PLCB3_HUMAN'

't25553aep|TCB1_CAEBR'

In [15]:

```
photo_all <- c(
  't12724aep-GNAS-HOMAM',
  't15699aep-GNAO-BOVIN',
  't12031aep-GNAI-PATPE',
  't13138aep-GBG2-PONAB',
  't26652aep-GBG7-HUMAN',
  't13671aep-GRK5-BOVIN',
  't26867aep-PDE11-MOUSE',
  't38455aep-PDE11-DROME',
  't20025aep-PDE6-DROMO',
  't25255aep-PDE6D-MOUSE',
  't6798aep-PDE9A-PANTR',
  't27655aep-CNGA3-HUMAN',
  't26213aep-RGS12-RAT',
  't7063aep-GCY-STRPU',
  't21324aep-GC76C-DROME',
  't38370aep-NCAH-DROME',
  't612aep-GNAQ-MIZYE',
  't29781aep-PIP1-DROME',
  't27547aep-ANK3-RAT',
  't19432aep-GBB-PINFU',
  't17074aep-ARBK2-BOVIN',
  't14420aep-ARRB1-MACFA',
  't7389aep-S14L1-HUMAN',
  't15272aep',
  't911aep-ANR28-HUMAN',
  't27546aep-ANR28-MOUSE',
  't11518aep-ANK1-HUMAN',
  't32411aep-CNGA2-BOVIN',
  't36031aep-ANPRA-MOUSE',
  't28209aep',
  't2619aep-PIP1-DROME',
  't30156aep-PLCB3-HUMAN',
  't25553aep-TCB1-CAEBR'
)

DotPlot(
  object = hydraV3,
  features = photo_all,
  cols = c("lightgrey", "black"),
  dot.scale = 6,
  dot.min = 0.05,  # setting percent expression threshold
```

```
  cluster.idents = FALSE,
  scale = FALSE
) + RotatedAxis()

# Resize plot
options(repr.plot.width = 15, repr.plot.height = 10)
```

```
Warning message:
“Removed 474 rows containing missing values or values outside the scale range
(`geom_point()`).”
```

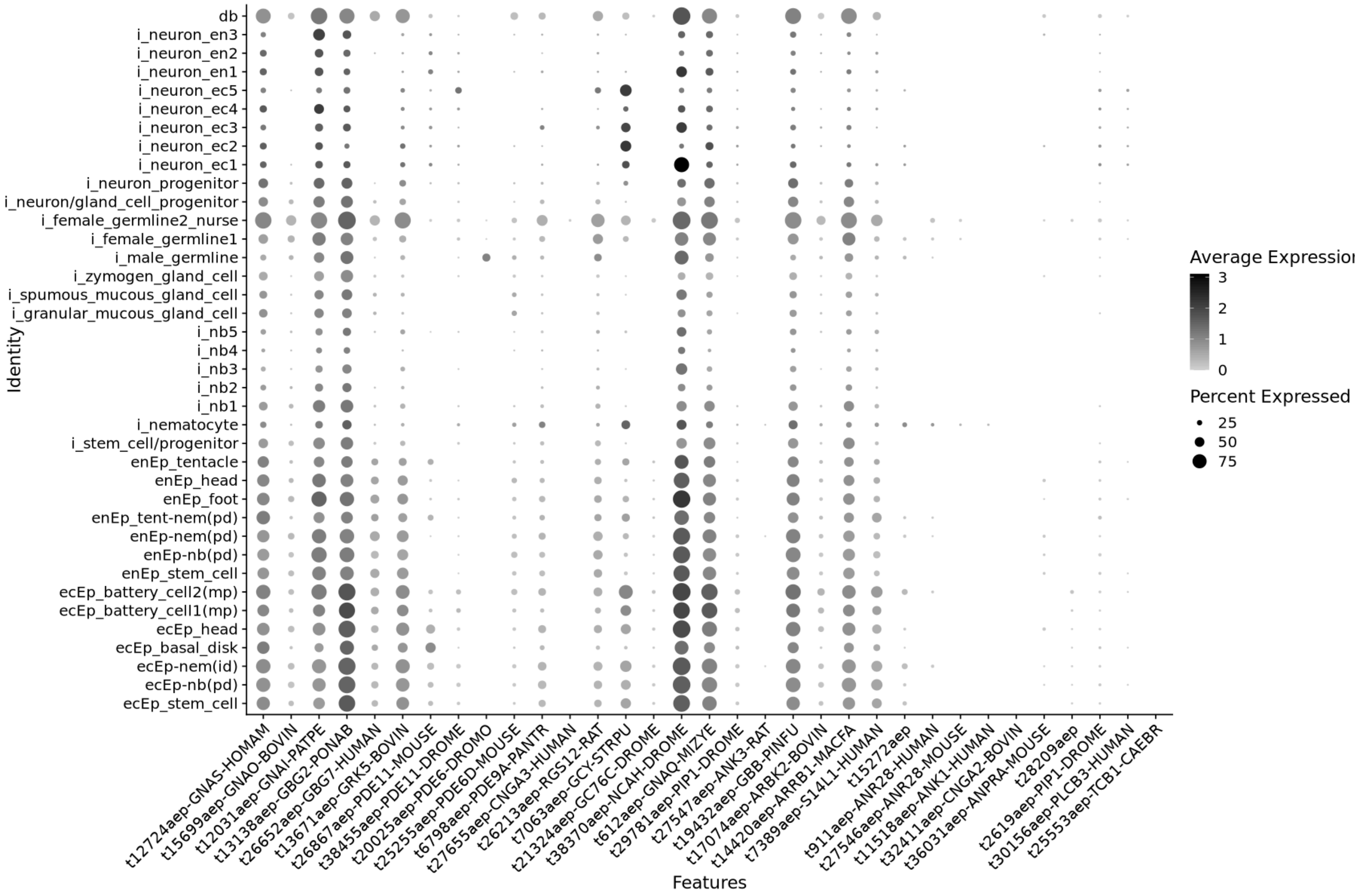

Wnt

In [16]:
```
# WNT3, pulled from HGP Genome Blast with Human WNT3 as bait sequence (GenBank: AAI03922
grep("t14194aep", rownames(interstitial@logupx.data), value=T)
```

't14194aep|WNT3_MOUSE'

In [17]:
```
# Export opsin plot on interstitial cell lineage
#pdf("/Users/marina/Desktop/Bioinformatics/Projects/Worster_Hydra_Notebook/opsin_URD_and
plotTree(interstitial,
         "t14194aep|WNT3_MOUSE",
          tree.size = 2,
          cell.size = 1,
         )
#dev.off()

# Export opsin plot on ectodermal cell lineage
#pdf("/Users/marina/Desktop/Bioinformatics/Projects/Worster_Hydra_Notebook/opsin_URD_and
plotTree(ectoderm,
         "t14194aep|WNT3_MOUSE",
          tree.size = 2,
          cell.size = 1,
```

```r
        )
#dev.off()

# Export opsin plot on endodermal lineage
#pdf("/Users/marina/Desktop/Bioinformatics/Projects/Worster_Hydra_Notebook/opsin_URD_and
plotTree(endoderm,
        "t14194aep|WNT3_MOUSE",
         tree.size = 2,
         cell.size = 1)
#dev.off()

plotSmoothFitMultiCascade(ectoderm.splines, c("t14194aep|WNT3_MOUSE"))

DotPlot(
  object = hydraV3,
  features = "t14194aep-WNT3-MOUSE",
  cols = c("lightgrey", "black"),
  dot.scale = 6,
  dot.min = 0.05,  # setting percent expression threshold
  cluster.idents = FALSE,
  scale = FALSE
) + RotatedAxis()
```

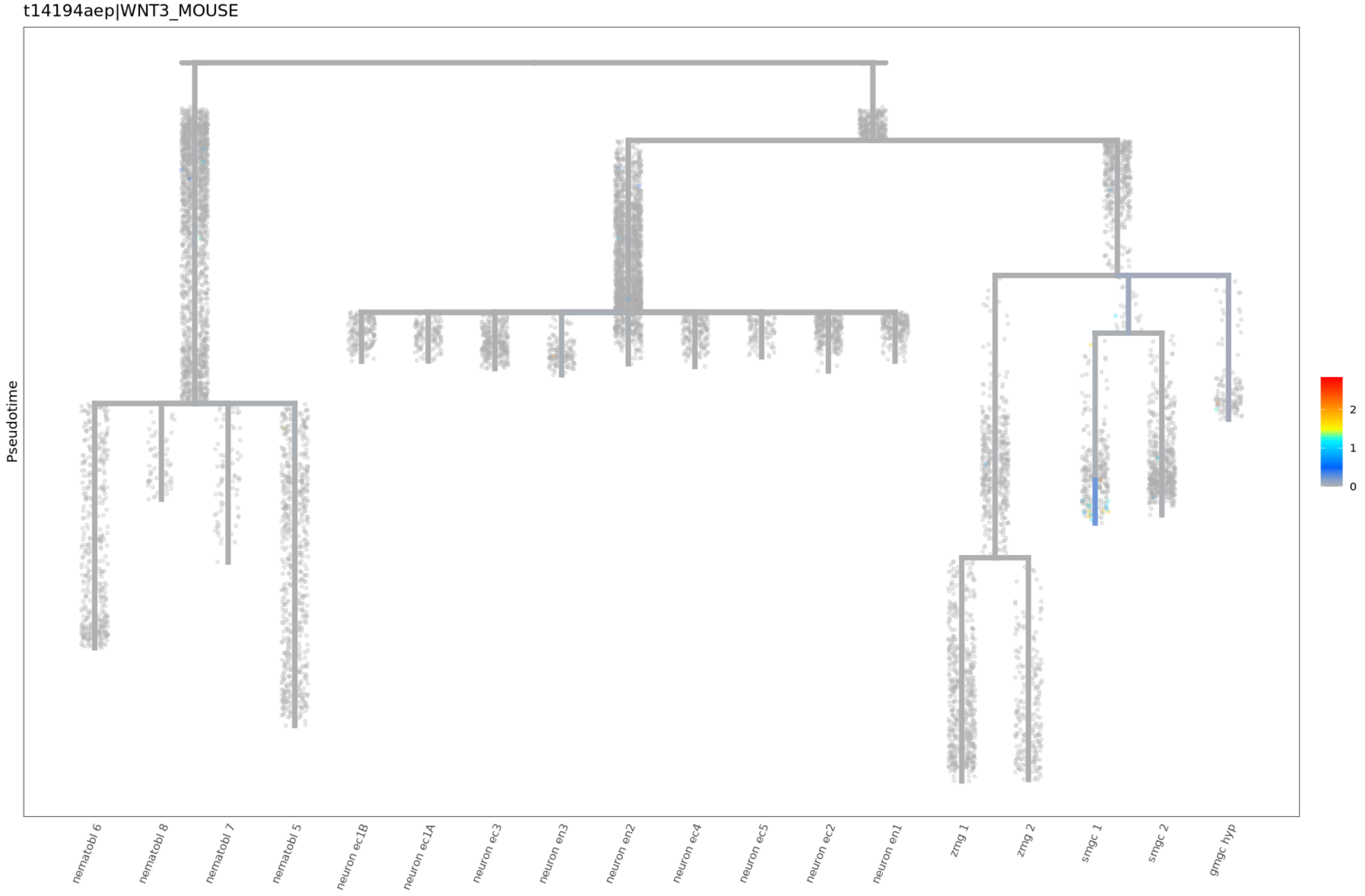

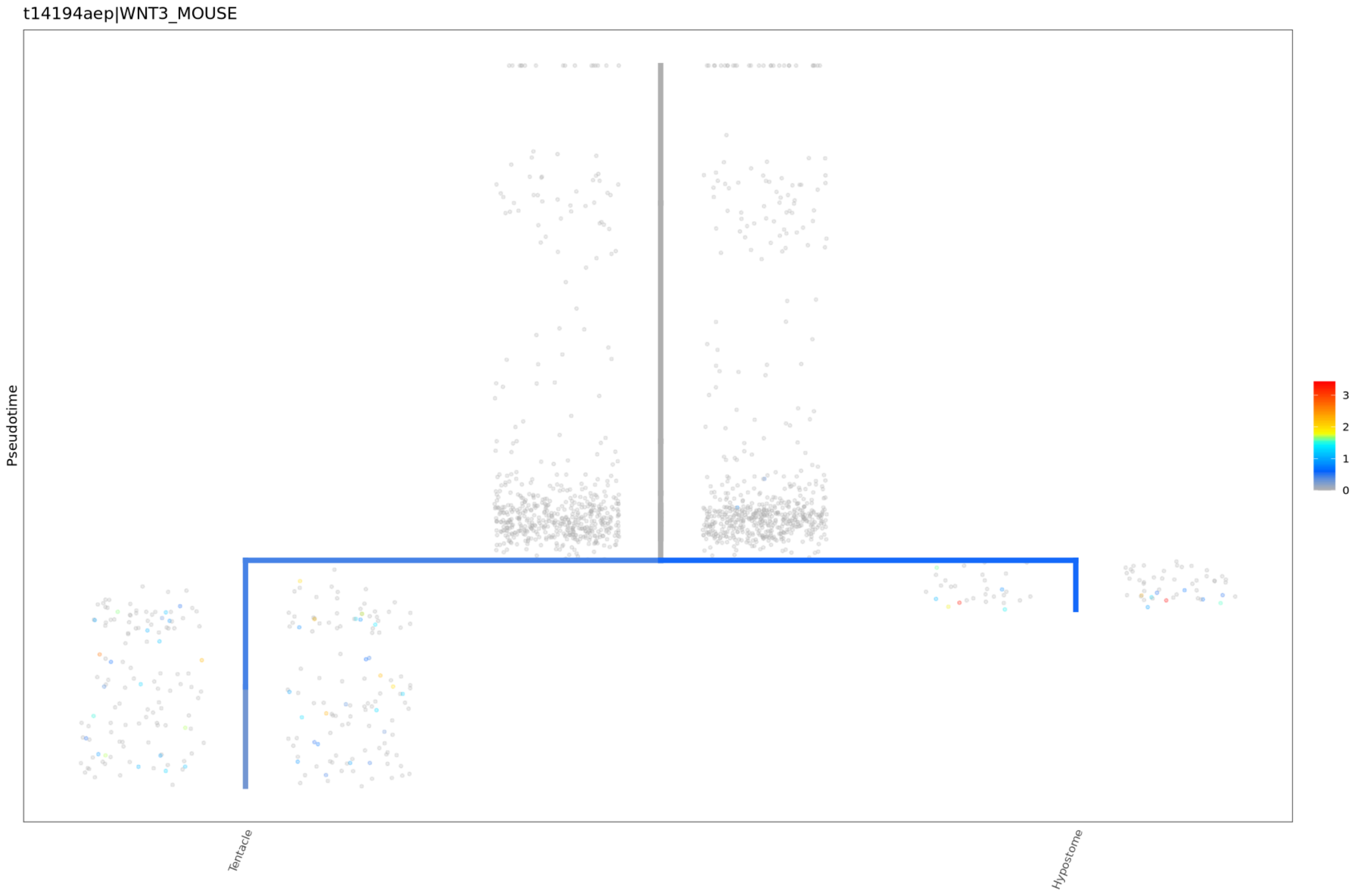

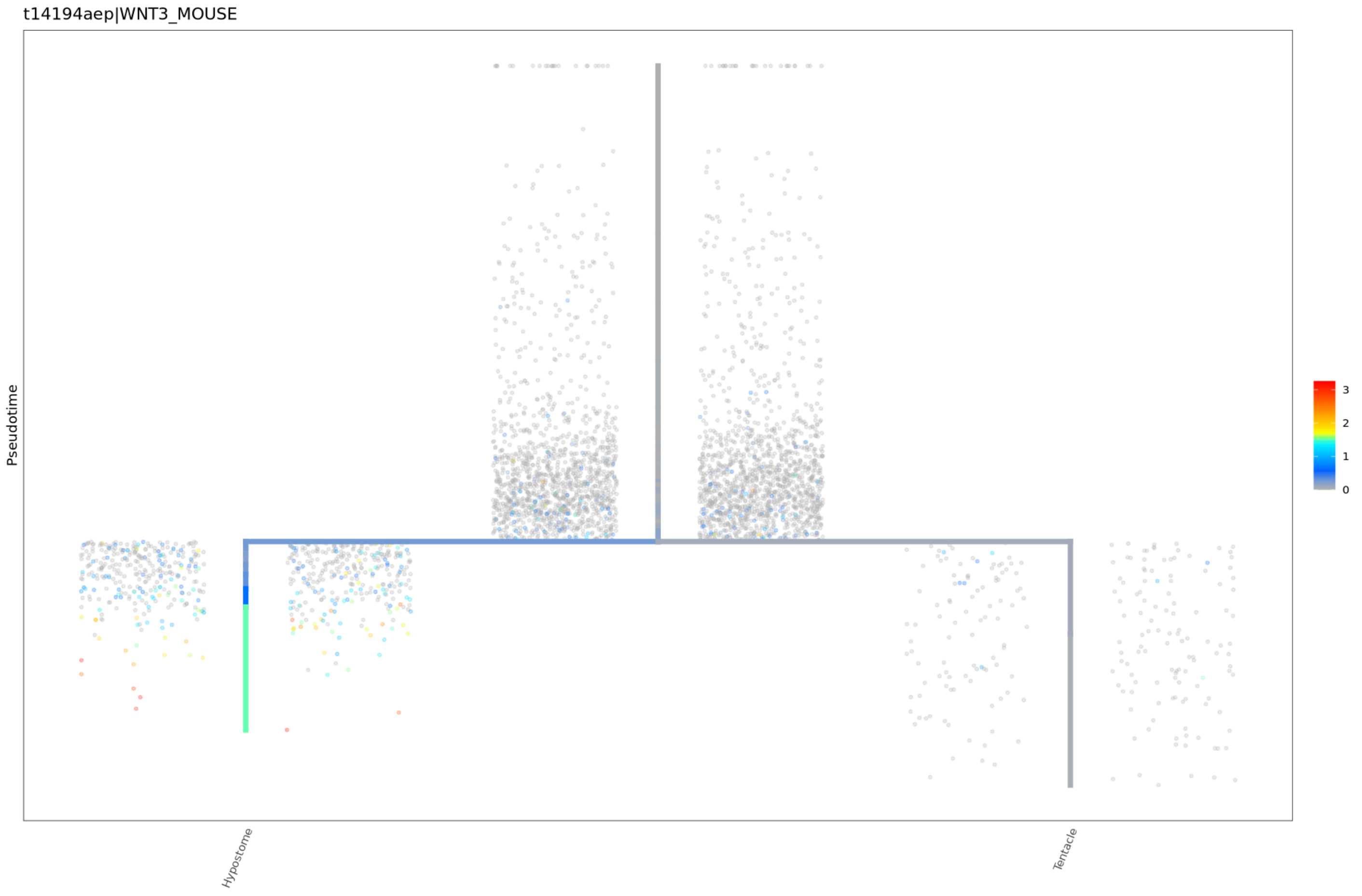


```
Warning message:
“Removed 31 rows containing missing values or values outside the scale range
(`geom_point()`).”
```

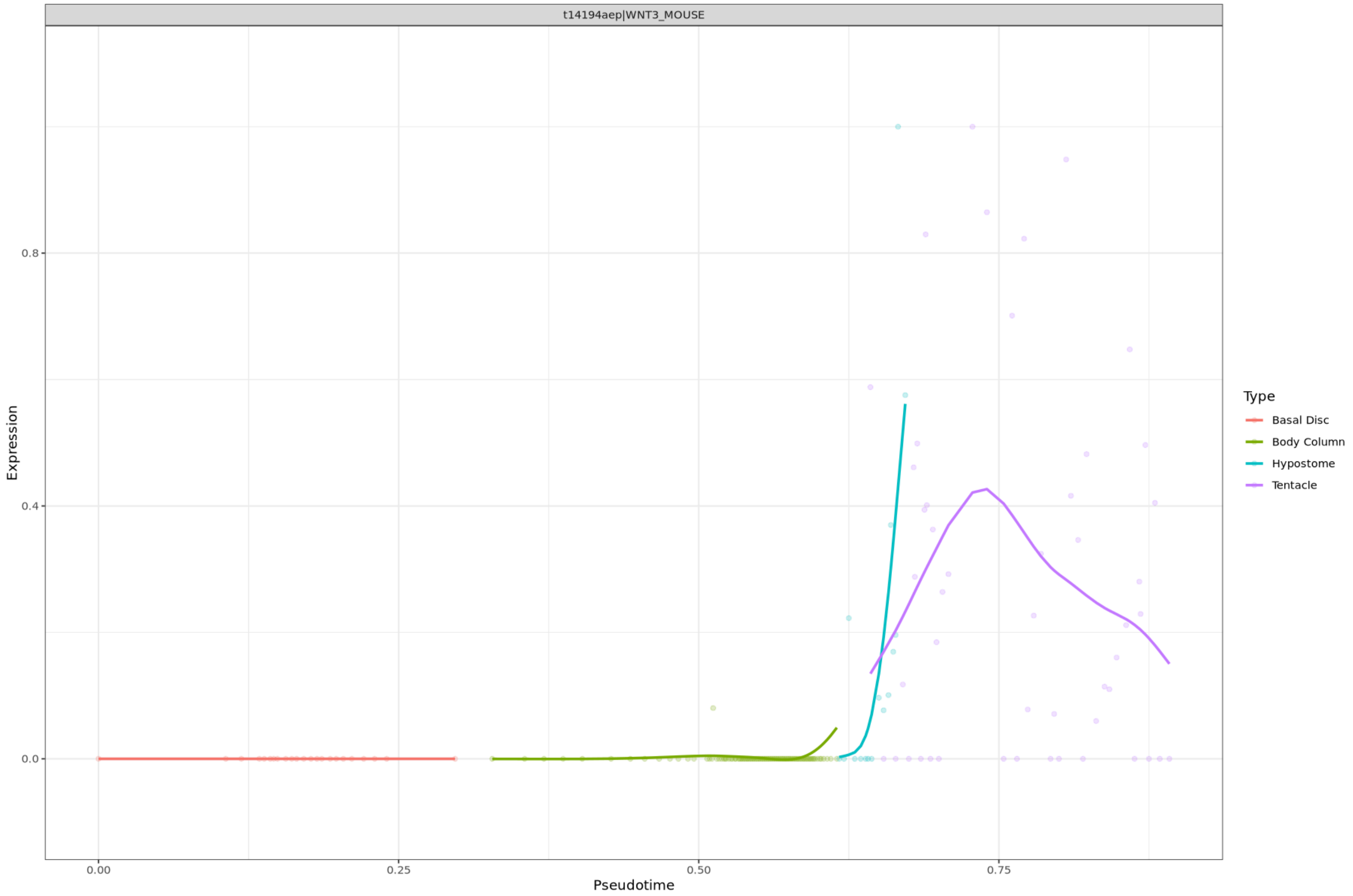

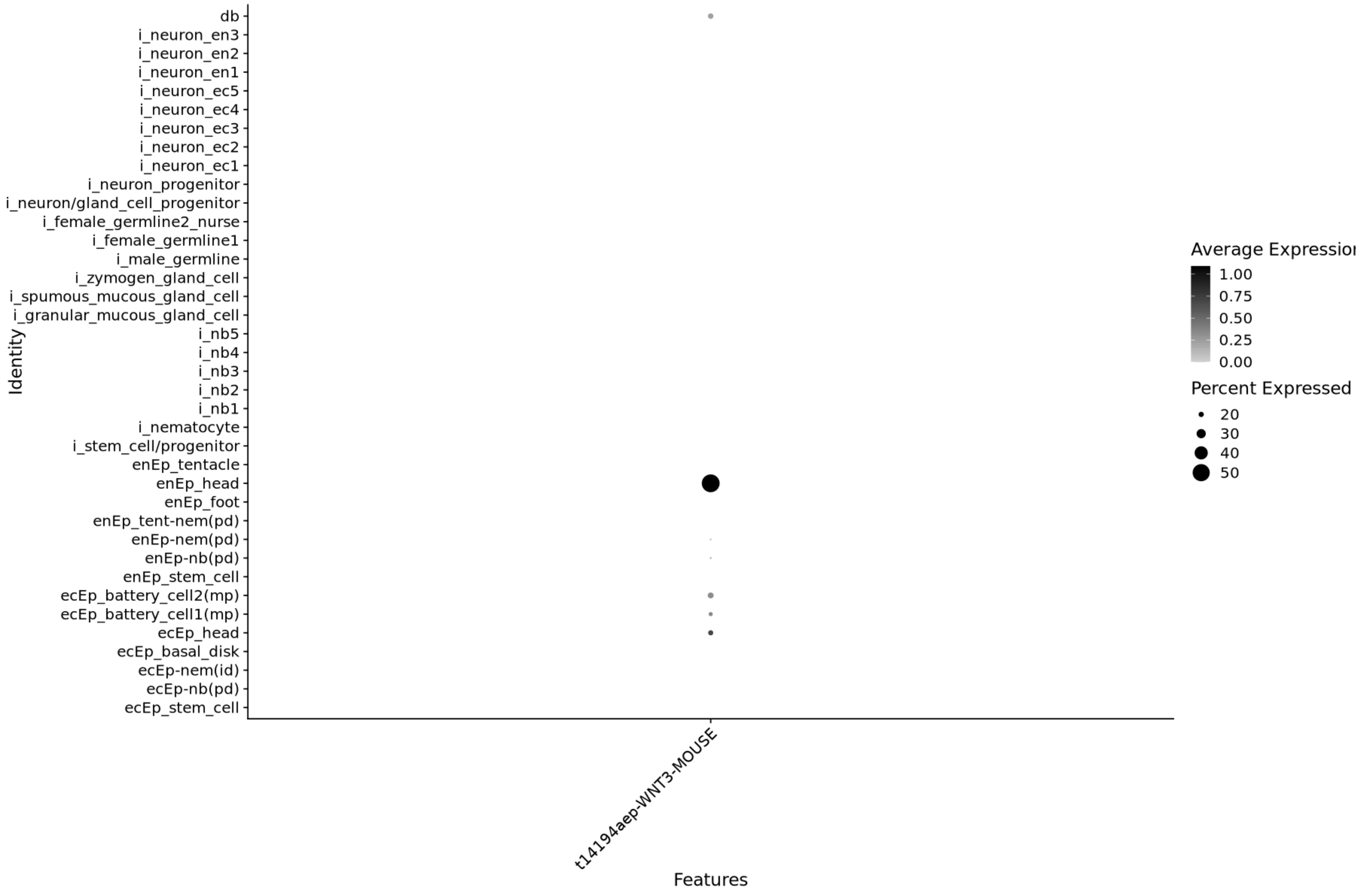

Pulling Average Expression Values

In [18]:
```
avg_expr <- AverageExpression(
  hydraV3,
```

```r
  assays = "RNA",
  features = new.hydraV3.features,
  return.seurat = FALSE,
  group.by = "ident",
  add.ident = NULL,
  layer = "data",  #data : normalized UMI counts , scaled.data : zscores of data
  verbose = TRUE
)
print (avg_expr)
# Save the average expression result as CSV
write.csv(avg_expr$RNA, file = "./output/hydraV3_average_expression.csv", row.names = TR
```

```
As of Seurat v5, we recommend using AggregateExpression to perform pseudo-bulk analysis.
This message is displayed once per session.
Names of identity class contain underscores ('_'), replacing with dashes ('-')
This message is displayed once every 8 hours.
Warning message:
“The following 2 features were not found in the RNA assay: t37969aep, t24989aep”
```

```
$RNA
43 x 38 sparse Matrix of class "dgCMatrix"
```

```
  [[ suppressing 38 column names ‘ecEp-stem-cell’, ‘ecEp-nb(pd)’, ‘ecEp-nem(id)’ ... ]]
```

t20729aep-OPSP-COLLI	.	.	.	.
t20043aep-OPSX-HUMAN	3.011149e-02	0.0324250400	0.0387039525	0.0020758016
t20044aep	.	.	.	.
t29150aep-OPSX-HUMAN	6.194823e-02	0.0779737214	0.0654059707	0.0227557692
t25412aep-OPSO-SALSA	6.749890e-04	0.0005667497	0.0013937243	0.0081272023
t3168aep-OPN3-HUMAN	8.542744e-04	.	0.0019554651	.
t3233aep	3.038523e-03	0.0192116010	0.0051393376	.
t32881aep-OPN3-MOUSE	4.616382e-03	0.0119607799	0.0042774072	0.0029832651
t3169aep-OPSX-HUMAN	.	.	0.0008104995	.
t21413aep-OPN4-PHOSU	1.614970e-03	0.0036380886	0.0004579642	0.0055775155
t24564aep-OPSR-CAPHI	.	0.0006724458	.	0.0039855691
t31971aep	4.248522e-04	0.0003635349	.	.
t355aep-OPSG3-DANRE	1.718603e-04	0.0006938349	0.0010857366	.
t10575aep-OPSR-BOVIN	6.319976e-04	.	.	0.0021038317
t24044aep-OPSP-ICTPU	1.685853e-03	.	0.0138836977	.
t26793aep-OPSO-RUTRU	8.267352e-04	0.0008573717	.	.
t9221aep-OPN3-HUMAN	1.828216e-03	0.0009944242	0.0075588161	0.0017158286
t4885aep-OPN5-MOUSE	1.127630e-04	.	0.0015677682	0.0016914292
t15588aep-OPN3-HUMAN	.	.	.	.
t27882aep-OPN3-HUMAN	8.291038e-04	0.0036095143	.	.
t33805aep-OPN4B-XENLA	2.693103e-03	0.0008104164	0.0015668005	.
t3337aep	1.376763e-03	0.0011610807	.	.
t36346aep-OPN3-MOUSE	6.490575e-03	0.0058883217	0.0032610227	0.0165442705
t33516aep-OPSD2-MIZYE	.	.	.	.
t33517aep-OPSD2-MIZYE	3.789271e-03	0.0029025990	0.0015840480	.
t4602aep	2.614205e-04	0.0057801769	0.0067721562	.
t26465aep-OPN3-HUMAN	.	0.0023519478	.	.
t26466aep-OPN3-HUMAN	.	0.0005922384	.	.
t29512aep-OPSP-CHICK	2.448179e-03	0.0010091235	.	0.0097220512
t33568aep	7.016290e-04	0.0014534176	0.0016774659	0.0040286528
t14044aep-OPSD2-MIZYE	1.220890e-03	0.0017274221	.	0.0109978092
t29959aep-OPN4B-XENLA	2.101342e-01	0.1479241196	0.1694292553	0.1072197434
t17353aep-OPN4B-XENLA	5.246356e-03	0.0029591694	0.0196249993	0.0005521449
t2106aep-OPN3-HUMAN	3.705205e-04	0.0022112268	0.0004655361	.
t21092aep-OPSD-LOLFO	1.958290e-02	0.0173452320	0.0129463186	0.0351483863
t27688aep-OPSP-COLLI	3.593230e-04	0.0020932431	0.0107404735	0.0069125897
t36136aep-OPSD-LOLFO	3.921379e-04	.	.	.
t36280aep-OPS2-DROPS	8.156294e-05	0.0003019418	.	.
t4128aep-OPSD2-MIZYE	2.653569e-03	0.0003635349	.	0.0204143673
t31375aep-OPSC1-HEMSA	8.237775e-04	0.0033811616	0.0005313923	.
t20210aep	.	.	.	.
t16278aep-OPSC2-HEMSA	9.054037e-04	.	0.0008293939	.
t32850aep	1.872597e-04	0.0036011030	.	.

t20729aep-OPSP-COLLI	0.0020686069	.	.	.
t20043aep-OPSX-HUMAN	0.0378553897	0.018823805	0.0329805042	9.676325e-05
t20044aep	.	.	.	.
t29150aep-OPSX-HUMAN	0.0686275277	0.015506816	0.0239534459	1.250087e-03
t25412aep-OPSO-SALSA	0.0046176945	.	0.0018164290	4.520028e-03
t3168aep-OPN3-HUMAN	0.0012162713	.	.	.
t3233aep	0.0026710604	.	0.0130750354	8.247301e-04
t32881aep-OPN3-MOUSE	0.0046993032	.	.	2.157690e-04
t3169aep-OPSX-HUMAN	.	.	.	.
t21413aep-OPN4-PHOSU	0.0032237992	.	0.0407060278	5.134958e-03
t24564aep-OPSR-CAPHI	0.0004337933	0.017131256	0.0046616017	1.633672e-04
t31971aep	.	.	.	.
t355aep-OPSG3-DANRE	.	.	.	2.323759e-04
t10575aep-OPSR-BOVIN	0.0027462292	.	.	6.865764e-04
t24044aep-OPSP-ICTPU	.	.	0.0003847068	4.349206e-04
t26793aep-OPSO-RUTRU	0.0015249322	0.001093231	0.0032447437	4.590518e-04
t9221aep-OPN3-HUMAN	0.0039902311	0.002497877	.	1.131687e-03

t4885aep-OPN5-MOUSE	.	.	.	9.189838e-05
t15588aep-OPN3-HUMAN	.	.	.	.
t27882aep-OPN3-HUMAN	.	.	.	4.492133e-04
t33805aep-OPN4B-XENLA	0.0051644698	.	0.0035816224	3.855624e-03
t3337aep	.	.	.	5.009653e-04
t36346aep-OPN3-MOUSE	0.0241135157	0.003383637	0.0143031196	1.865978e-03
t33516aep-OPSD2-MIZYE	.	.	.	.
t33517aep-OPSD2-MIZYE	0.0018601313	.	0.0006802617	.
t4602aep	.	0.001974412	0.0112844464	3.340283e-03
t26465aep-OPN3-HUMAN	.	.	0.0048892763	2.801042e-04
t26466aep-OPN3-HUMAN	.	.	.	2.823131e-04
t29512aep-OPSP-CHICK	0.0035503329	.	0.0014895076	4.378218e-04
t33568aep	.	.	0.0069120177	.
t14044aep-OPSD2-MIZYE	.	.	0.0012293532	6.447473e-04
t29959aep-OPN4B-XENLA	0.0187728079	0.022351363	0.0028315535	1.426221e-02
t17353aep-OPN4B-XENLA	0.0233667010	0.003308659	0.0125754160	3.870338e-03
t2106aep-OPN3-HUMAN	0.0081157463	0.002806781	.	7.010136e-04
t21092aep-OPSD-LOLFO	.	.	0.0019559944	2.740137e-04
t27688aep-OPSP-COLLI	0.0036499255	.	0.0632411463	8.764724e-02
t36136aep-OPSD-LOLFO	.	.	0.0076952869	2.275645e-03
t36280aep-OPS2-DROPS	0.0012061773	.	.	1.736838e-04
t4128aep-OPSD2-MIZYE	.	0.016954900	0.0122198374	4.010437e-04
t31375aep-OPSC1-HEMSA	0.0003495406	0.007647599	.	1.438211e-03
t20210aep	.	.	.	2.273610e-04
t16278aep-OPSC2-HEMSA	0.0004696902	.	.	1.962208e-03
t32850aep	0.0019656406	.	0.0021734908	2.178397e-04

t20729aep-OPSP-COLLI	.	.	.	0.0009294026
t20043aep-OPSX-HUMAN	.	0.0026181204	0.155083552	0.0006272272
t20044aep	.	.	0.014875839	.
t29150aep-OPSX-HUMAN	.	.	0.041357541	0.0827378166
t25412aep-OPSO-SALSA	.	.	.	0.0006323502
t3168aep-OPN3-HUMAN	.	.	.	.
t3233aep	.	0.0023559279	.	.
t32881aep-OPN3-MOUSE	.	0.0008447237	.	.
t3169aep-OPSX-HUMAN	.	0.0015216654	.	0.0006209136
t21413aep-OPN4-PHOSU	0.002179050	.	0.003586623	0.0104102358
t24564aep-OPSR-CAPHI	.	.	.	.
t31971aep	.	.	.	.
t355aep-OPSG3-DANRE	.	.	.	0.0025392769
t10575aep-OPSR-BOVIN	.	.	0.019833899	0.0011717766
t24044aep-OPSP-ICTPU	.	.	.	.
t26793aep-OPSO-RUTRU	.	.	.	.
t9221aep-OPN3-HUMAN	.	0.0072284053	.	0.0019302373
t4885aep-OPN5-MOUSE	.	.	.	.
t15588aep-OPN3-HUMAN	.	.	.	.
t27882aep-OPN3-HUMAN	.	0.0013032289	.	0.0014925851
t33805aep-OPN4B-XENLA	.	0.0061062125	.	0.0030814428
t3337aep	.	.	.	0.0020265100
t36346aep-OPN3-MOUSE	0.007285141	0.0092835925	.	0.0096841739
t33516aep-OPSD2-MIZYE	.	.	.	0.0007156436
t33517aep-OPSD2-MIZYE	.	.	.	.
t4602aep	0.005992608	0.0128139182	0.011588023	0.0464049807
t26465aep-OPN3-HUMAN	.	0.0018609414	.	0.0063686323
t26466aep-OPN3-HUMAN	.	0.0035213127	0.003586623	0.0049248708
t29512aep-OPSP-CHICK	0.006663815	.	.	0.0162113085
t33568aep	0.002070407	.	.	.
t14044aep-OPSD2-MIZYE	0.004732679	.	.	.
t29959aep-OPN4B-XENLA	0.005256491	0.0163085855	.	0.0739051333
t17353aep-OPN4B-XENLA	.	0.0084698533	0.006403678	0.0042775561
t2106aep-OPN3-HUMAN	0.003004772	0.0023143107	.	0.0016683193
t21092aep-OPSD-LOLFO	.	.	.	.

t27688aep-OPSP-COLLI	0.098378927	0.1436478977	0.052522273	0.0303187225
t36136aep-OPSD-LOLFO	.	0.0018293609	0.016015229	0.0009587734
t36280aep-OPS2-DROPS	.	.	.	.
t4128aep-OPSD2-MIZYE	.	0.0018129953	0.026134627	0.0061890206
t31375aep-OPSC1-HEMSA	0.008168066	.	.	.
t20210aep	.	.	.	0.0031692467
t16278aep-OPSC2-HEMSA	0.005269787	.	.	0.0085736848
t32850aep	0.005024073	.	.	.

t20729aep-OPSP-COLLI	.	.	0.0076460508	.
t20043aep-OPSX-HUMAN	0.002009152	0.0353612652	.	.
t20044aep	.	0.0171158838	.	.
t29150aep-OPSX-HUMAN	0.005268568	0.0013452074	0.0014463627	0.007479080
t25412aep-OPSO-SALSA	.	.	0.0021518891	.
t3168aep-OPN3-HUMAN	.	.	.	0.052168507
t3233aep	.	0.0040336341	.	.
t32881aep-OPN3-MOUSE	.	.	.	0.001634506
t3169aep-OPSX-HUMAN	.	.	.	0.018419749
t21413aep-OPN4-PHOSU	0.015193336	0.0027170310	0.0021060466	0.086281227
t24564aep-OPSR-CAPHI	.	0.0033725766	0.0005469661	0.009531625
t31971aep	.	.	.	0.008555275
t355aep-OPSG3-DANRE	.	.	.	0.003544451
t10575aep-OPSR-BOVIN	.	0.0021118156	.	.
t24044aep-OPSP-ICTPU	.	0.0048867639	.	0.021307727
t26793aep-OPSO-RUTRU	.	.	.	.
t9221aep-OPN3-HUMAN	.	0.0006404829	.	.
t4885aep-OPN5-MOUSE	.	.	0.0008185158	.
t15588aep-OPN3-HUMAN	.	.	0.0001533491	.
t27882aep-OPN3-HUMAN	.	.	.	0.003130660
t33805aep-OPN4B-XENLA	0.002809163	0.0053818243	0.0002328992	.
t3337aep	.	.	.	.
t36346aep-OPN3-MOUSE	0.004698485	0.0012037745	0.0001793603	0.026897786
t33516aep-OPSD2-MIZYE	.	.	0.0060961064	.
t33517aep-OPSD2-MIZYE	0.005691944	.	.	.
t4602aep	.	0.0121295212	0.0010073783	0.049676307
t26465aep-OPN3-HUMAN	.	0.0005040879	.	0.014716550
t26466aep-OPN3-HUMAN	.	.	.	.
t29512aep-OPSP-CHICK	0.002589715	.	0.0011281288	0.013460969
t33568aep	.	.	.	.
t14044aep-OPSD2-MIZYE	0.001761292	0.0097735278	.	.
t29959aep-OPN4B-XENLA	0.002126902	0.0087163983	0.0017485968	0.015003871
t17353aep-OPN4B-XENLA	0.003024003	0.0193082531	.	0.631750659
t2106aep-OPN3-HUMAN	0.001547076	.	0.0077520125	0.048607420
t21092aep-OPSD-LOLFO	.	.	0.0012762541	0.017788024
t27688aep-OPSP-COLLI	0.077462665	0.0097009863	0.0002747963	0.433738333
t36136aep-OPSD-LOLFO	0.010949086	0.0410245777	.	.
t36280aep-OPS2-DROPS	.	.	.	.
t4128aep-OPSD2-MIZYE	.	0.0022003488	0.0013521290	0.035561175
t31375aep-OPSC1-HEMSA	.	.	0.0111275694	.
t20210aep	0.000729666	0.0046330697	.	.
t16278aep-OPSC2-HEMSA	.	0.0017043214	0.0013626970	.
t32850aep	.	.	0.0111060808	.

t20729aep-OPSP-COLLI	.	.	.	.
t20043aep-OPSX-HUMAN	.	.	.	.
t20044aep	.	.	.	.
t29150aep-OPSX-HUMAN	0.0044630355	.	.	0.006306005
t25412aep-OPSO-SALSA	.	0.0011290841	0.001151956	.
t3168aep-OPN3-HUMAN	0.0003401907	.	.	.
t3233aep	.	.	.	.
t32881aep-OPN3-MOUSE	.	.	.	.
t3169aep-OPSX-HUMAN	.	.	.	.

t21413aep-OPN4-PHOSU	.	0.0022112622	.	0.027684856
t24564aep-OPSR-CAPHI	.	.	.	.
t31971aep	.	.	.	.
t355aep-OPSG3-DANRE	.	.	.	.
t10575aep-OPSR-BOVIN	0.0015137185	.	.	.
t24044aep-OPSP-ICTPU	.	.	0.003697886	.
t26793aep-OPSO-RUTRU	.	.	.	.
t9221aep-OPN3-HUMAN	0.0032312662	.	.	.
t4885aep-OPN5-MOUSE	.	.	.	.
t15588aep-OPN3-HUMAN	.	0.0019182842	.	.
t27882aep-OPN3-HUMAN	0.0005594991	0.0022020844	.	.
t33805aep-OPN4B-XENLA	.	0.0075007949	.	.
t3337aep	.	.	0.006934553	.
t36346aep-OPN3-MOUSE	0.0042904313	0.0021296605	.	.
t33516aep-OPSD2-MIZYE	.	.	.	.
t33517aep-OPSD2-MIZYE	.	.	.	.
t4602aep	0.0065176217	.	0.016495281	0.077351251
t26465aep-OPN3-HUMAN	.	.	.	.
t26466aep-OPN3-HUMAN	.	.	.	.
t29512aep-OPSP-CHICK	.	0.0033552363	.	.
t33568aep	.	.	.	.
t14044aep-OPSD2-MIZYE	.	.	.	.
t29959aep-OPN4B-XENLA	.	0.0006908627	0.001142073	.
t17353aep-OPN4B-XENLA	.	.	0.008731451	.
t2106aep-OPN3-HUMAN	0.0161887306	.	.	.
t21092aep-OPSD-LOLFO	0.0011345144	.	0.001142073	.
t27688aep-OPSP-COLLI	.	0.0197447400	0.003518448	.
t36136aep-OPSD-LOLFO	.	.	.	.
t36280aep-OPS2-DROPS	.	.	.	.
t4128aep-OPSD2-MIZYE	.	.	.	0.010028340
t31375aep-OPSC1-HEMSA	0.0040565321	0.0517681828	0.155724815	.
t20210aep	.	.	.	.
t16278aep-OPSC2-HEMSA	.	.	.	.
t32850aep	0.0023549544	0.0086460819	0.112894451	.

t20729aep-OPSP-COLLI	.	.	.	.
t20043aep-OPSX-HUMAN	0.009043161	.	0.0004289834	0.0064242415
t20044aep	.	.	.	0.0025868730
t29150aep-OPSX-HUMAN	0.004140875	0.0027015345	.	.
t25412aep-OPSO-SALSA	.	.	.	0.0007840982
t3168aep-OPN3-HUMAN	.	.	.	.
t3233aep	.	.	.	.
t32881aep-OPN3-MOUSE	.	.	0.0027346673	.
t3169aep-OPSX-HUMAN	.	.	.	.
t21413aep-OPN4-PHOSU	.	0.6979306478	0.5974657660	0.0246753093
t24564aep-OPSR-CAPHI	.	.	.	.
t31971aep	.	.	.	.
t355aep-OPSG3-DANRE	.	.	.	.
t10575aep-OPSR-BOVIN	.	.	.	.
t24044aep-OPSP-ICTPU	.	.	.	.
t26793aep-OPSO-RUTRU	.	.	.	.
t9221aep-OPN3-HUMAN	0.041188900	0.0026666471	0.0067020850	.
t4885aep-OPN5-MOUSE	.	0.0079897731	.	.
t15588aep-OPN3-HUMAN	.	.	0.0044379020	0.0034921733
t27882aep-OPN3-HUMAN	.	0.0956890637	0.1531229493	0.0600691581
t33805aep-OPN4B-XENLA	0.001091354	.	.	.
t3337aep	.	0.0053625054	.	.
t36346aep-OPN3-MOUSE	.	0.0185442641	0.0255294000	0.0043633708
t33516aep-OPSD2-MIZYE	.	.	.	.
t33517aep-OPSD2-MIZYE	.	.	.	.
t4602aep	0.428510391	.	.	.
t26465aep-OPN3-HUMAN	0.123669999	.	.	.

t26466aep-OPN3-HUMAN	0.039056713	.	.	0.0005847172
t29512aep-OPSP-CHICK	.	0.0136314068	0.0027658675	.
t33568aep	.	.	.	.
t14044aep-OPSD2-MIZYE	.	.	.	0.0003537292
t29959aep-OPN4B-XENLA	0.027024257	.	0.0158698002	0.0037086394
t17353aep-OPN4B-XENLA	0.004331389	.	.	0.0018530749
t2106aep-OPN3-HUMAN	.	0.0096873782	0.0064142384	0.0037365103
t21092aep-OPSD-LOLFO	.	0.0005016051	.	.
t27688aep-OPSP-COLLI	0.022444472	.	0.0052235128	0.0026656978
t36136aep-OPSD-LOLFO	.	.	.	0.0009271428
t36280aep-OPS2-DROPS	.	.	.	0.0004717565
t4128aep-OPSD2-MIZYE	.	.	0.0026232529	.
t31375aep-OPSC1-HEMSA	.	0.0027464077	.	0.0017135908
t20210aep	.	0.0106080702	.	0.0104726289
t16278aep-OPSC2-HEMSA	.	0.0078821489	0.0077706421	0.0044048515
t32850aep	.	.	.	0.0016277528

t20729aep-OPSP-COLLI	.	.	.	0.0275903857
t20043aep-OPSX-HUMAN	0.003421488	0.0084480009	.	0.0009429365
t20044aep	.	.	.	.
t29150aep-OPSX-HUMAN	.	0.0105289793	0.0017306643	.
t25412aep-OPSO-SALSA	0.006971872	.	.	.
t3168aep-OPN3-HUMAN	.	.	0.0007470255	.
t3233aep	.	0.0069872744	0.0145331547	.
t32881aep-OPN3-MOUSE	.	0.0101622503	0.0146386107	.
t3169aep-OPSX-HUMAN	.	.	.	.
t21413aep-OPN4-PHOSU	0.005299572	.	0.0020581881	0.0311651483
t24564aep-OPSR-CAPHI	.	.	.	0.0010520570
t31971aep	.	.	0.0006535383	.
t355aep-OPSG3-DANRE	.	.	.	.
t10575aep-OPSR-BOVIN	0.008169401	.	.	.
t24044aep-OPSP-ICTPU	.	.	.	.
t26793aep-OPSO-RUTRU	.	.	0.0008465768	.
t9221aep-OPN3-HUMAN	.	.	0.0016205790	0.1147398435
t4885aep-OPN5-MOUSE	.	.	0.0007016537	.
t15588aep-OPN3-HUMAN	.	0.0005011844	.	.
t27882aep-OPN3-HUMAN	.	0.0035373863	.	0.0584342505
t33805aep-OPN4B-XENLA	.	.	.	0.0034506050
t3337aep	.	0.0003932604	.	.
t36346aep-OPN3-MOUSE	0.010044166	0.0008320916	.	0.0057748545
t33516aep-OPSD2-MIZYE	.	.	.	0.0248826555
t33517aep-OPSD2-MIZYE	.	.	.	.
t4602aep	.	.	.	.
t26465aep-OPN3-HUMAN	.	.	.	.
t26466aep-OPN3-HUMAN	0.025959209	.	.	.
t29512aep-OPSP-CHICK	0.012297901	0.0042899745	.	0.0148306923
t33568aep	.	.	0.0016781232	.
t14044aep-OPSD2-MIZYE	.	.	.	.
t29959aep-OPN4B-XENLA	0.010995238	0.0064953834	0.0069682270	0.0028074951
t17353aep-OPN4B-XENLA	.	0.0006778928	0.0011787460	0.0011807471
t2106aep-OPN3-HUMAN	0.003698650	.	0.0004679596	0.0017836972
t21092aep-OPSD-LOLFO	.	.	.	.
t27688aep-OPSP-COLLI	0.008381167	0.0004250566	.	.
t36136aep-OPSD-LOLFO	.	.	.	.
t36280aep-OPS2-DROPS	.	.	0.0004666694	.
t4128aep-OPSD2-MIZYE	.	0.0011189144	.	.
t31375aep-OPSC1-HEMSA	.	.	.	0.0069377634
t20210aep	0.013943744	0.0007395907	0.0017971900	.
t16278aep-OPSC2-HEMSA	.	.	.	.
t32850aep	.	.	0.0007169757	0.0206337867

t20729aep-OPSP-COLLI	0.030712489	0.018112989	.	0.15649023	.

t20043aep-OPSX-HUMAN	.	0.144328227	0.44482896	.	.
t20044aep	.	.	.	.	.
t29150aep-OPSX-HUMAN	.	0.010196722	.	.	.
t25412aep-OPSO-SALSA	0.008296848	0.551850180	.	0.21588347	0.24362837
t3168aep-OPN3-HUMAN	.	0.009123638	.	0.04059793	.
t3233aep	.	0.016967155	0.01377020	.	.
t32881aep-OPN3-MOUSE	.	.	0.01377020	.	.
t3169aep-OPSX-HUMAN	.	.	0.03200061	.	.
t21413aep-OPN4-PHOSU	0.012856464	0.137770501	0.03155430	0.14497891	0.23121039
t24564aep-OPSR-CAPHI	0.011051507	0.067082100	.	0.25094851	0.04048993
t31971aep	.	.	.	.	.
t355aep-OPSG3-DANRE	.	0.011175482	.	.	.
t10575aep-OPSR-BOVIN	.	0.177607862	.	0.03065328	.
t24044aep-OPSP-ICTPU	.	0.076479499	.	.	.
t26793aep-OPSO-RUTRU	.	0.104685202	.	0.11140544	0.09916950
t9221aep-OPN3-HUMAN	0.260484867	0.071864064	0.02240043	0.07536302	0.15187951
t4885aep-OPN5-MOUSE	.	.	.	0.04357640	.
t15588aep-OPN3-HUMAN	0.005188617	0.008928938	.	0.03543762	.
t27882aep-OPN3-HUMAN	0.011394609	.	.	0.17257023	.
t33805aep-OPN4B-XENLA	0.013459092	0.264604907	0.03069801	0.54586346	0.07921763
t3337aep	.	0.003208666	.	0.13647680	.
t36346aep-OPN3-MOUSE	0.004659874	0.996561223	0.04867014	0.37154320	0.04370266
t33516aep-OPSD2-MIZYE	0.025469255	0.003836512	.	0.07021964	.
t33517aep-OPSD2-MIZYE	.	0.046062966	.	.	.
t4602aep	.	0.009808018	.	0.04371585	.
t26465aep-OPN3-HUMAN	0.002791634	.	.	.	.
t26466aep-OPN3-HUMAN	.	.	.	.	.
t29512aep-OPSP-CHICK	0.092820982	0.263912482	.	0.46965159	0.10879161
t33568aep	.	0.305515771	.	0.29432708	.
t14044aep-OPSD2-MIZYE	0.003274713	0.325924810	.	0.21900400	0.02299152
t29959aep-OPN4B-XENLA	0.016597292	0.033902310	0.05436931	0.22039433	.
t17353aep-OPN4B-XENLA	0.015523670	.	0.07606516	1.25118663	0.12146978
t2106aep-OPN3-HUMAN	0.005521212	0.041974190	0.03613674	0.13133723	0.01760018
t21092aep-OPSD-LOLFO	0.004924789	0.191522381	0.02036403	0.03910923	.
t27688aep-OPSP-COLLI	0.003624481	0.008064958	.	.	0.02399307
t36136aep-OPSD-LOLFO	0.002904748	0.089689877	.	0.11982536	0.04464027
t36280aep-OPS2-DROPS	.	.	0.03274158	0.07362912	.
t4128aep-OPSD2-MIZYE	.	0.027743273	0.03055623	0.05802083	0.05223596
t31375aep-OPSC1-HEMSA	0.001277957	.	0.04293062	.	.
t20210aep	.	0.244327919	.	.	.
t16278aep-OPSC2-HEMSA	.	0.036831870	.	0.03552839	0.06294653
t32850aep	.	0.032790756	0.09368291	0.04133495	.

t20729aep-OPSP-COLLI	.	0.01855415	.	.	.
t20043aep-OPSX-HUMAN	0.15979344	.	.	.	.
t20044aep	.	.	.	.	.
t29150aep-OPSX-HUMAN	.	.	.	.	.
t25412aep-OPSO-SALSA	0.41583507	0.65415441	0.014250800	.	0.014850273
t3168aep-OPN3-HUMAN	.	.	.	.	.
t3233aep	0.02983294	.	.	.	.
t32881aep-OPN3-MOUSE	.	.	.	.	.
t3169aep-OPSX-HUMAN	.	.	.	.	.
t21413aep-OPN4-PHOSU	.	0.19950685	0.894839650	0.15305402	0.046796121
t24564aep-OPSR-CAPHI	0.09057971	.	0.013920578	.	.
t31971aep	0.45346904	.	.	.	.
t355aep-OPSG3-DANRE	0.38593062	.	.	.	.
t10575aep-OPSR-BOVIN	0.50875261	.	0.024331847	.	.
t24044aep-OPSP-ICTPU	.	.	.	.	.
t26793aep-OPSO-RUTRU	.	.	0.014250800	0.03944166	.
t9221aep-OPN3-HUMAN	0.09220122	0.11614458	0.464310130	0.27169981	0.042703138
t4885aep-OPN5-MOUSE	.	.	.	.	.
t15588aep-OPN3-HUMAN	.	0.01178246	.	0.03944166	.

```
t27882aep-OPN3-HUMAN  .          0.05691002 .           0.75237333 0.013440995
t33805aep-OPN4B-XENLA .          0.01752280 0.012290372 .          .
t3337aep              .          .          .           .          .
t36346aep-OPN3-MOUSE  .          0.33603046 0.514872461 0.18544911 0.074674806
t33516aep-OPSD2-MIZYE .          0.01855415 .           .          .
t33517aep-OPSD2-MIZYE .          0.08858962 .           .          .
t4602aep              0.06905864 0.01089239 .           .          .
t26465aep-OPN3-HUMAN  .          .          .           .          .
t26466aep-OPN3-HUMAN  0.02490040 .          .           .          .
t29512aep-OPSP-CHICK  0.13840021 0.04580390 .           0.08763167 .
t33568aep             0.01570352 .          .           .          .
t14044aep-OPSD2-MIZYE .          .          0.049776008 .          .
t29959aep-OPN4B-XENLA .          .          0.009514802 .          0.011701768
t17353aep-OPN4B-XENLA .          .          0.092363271 0.05214770 .
t2106aep-OPN3-HUMAN   0.00949848 0.11730664 0.052711887 .          .
t21092aep-OPSD-LOLFO  0.44989266 0.05965555 .           .          .
t27688aep-OPSP-COLLI  .          .          .           .          0.098254147
t36136aep-OPSD-LOLFO  .          0.03170503 .           .          0.003585227
t36280aep-OPS2-DROPS  .          .          .           .          .
t4128aep-OPSD2-MIZYE  1.44176059 .          .           .          .
t31375aep-OPSC1-HEMSA .          .          .           .          .
t20210aep             .          .          0.005695195 .          .
t16278aep-OPSC2-HEMSA 0.01258812 0.26088529 .           0.04091871 0.012552997
t32850aep             .          .          .           0.02343501 .
```

Pulling Metagene Scores

In [19]:

```
#Extract each metagene score for each opsin
gene_ids <- id_inventory$gene_ID
gene_ids_noNA <- gene_ids[gene_ids != ""]
gene_ids_noDup <- unique(gene_ids_noNA)
opsinMetagenes <- matrix(nrow = length(gene_ids_noDup), ncol = ncol(corResults.df))
rownames(opsinMetagenes) <- gene_ids_noDup
colnames(opsinMetagenes) <- colnames(corResults.df)
opsinMetagenes <- as.data.frame(opsinMetagenes)

for (i in 1:nrow(opsinMetagenes)) {
  match_rows <- grepl(gene_ids_noDup[i], rownames(corResults.df))

  # Check if there's at least one match
  if (any(match_rows)) {
    opsinMetagenes[i,] <- corResults.df[match_rows,]
  } else {
    # Handle the case where no match is found, e.g., skip or assign default values
    opsinMetagenes[i,] <- NA  # Or assign any default value you prefer
  }
}
```

In [20]:

```
# Extract the highest scoring metagene for each opsin
# Initialize an empty data frame to store the results
top_opsin_metagene <- data.frame(Row_Name = character(nrow(opsinMetagenes)),
                                 Top_Column = character(nrow(opsinMetagenes)),
                                 Max_Value = numeric(nrow(opsinMetagenes)),
                                 stringsAsFactors = FALSE)

# Loop over each row to find the column with the highest value
for (i in 1:nrow(opsinMetagenes)) {
  # Ensure that only numeric columns are considered
  numeric_data <- opsinMetagenes[i, sapply(opsinMetagenes, is.numeric)]
```

```
  # If there are any numeric values, find the column with the max value
  if (length(numeric_data) > 0 && any(!is.na(numeric_data))) {
    # Check if 'wg' is present in the numeric columns
    if ("wg" %in% colnames(numeric_data)) {
      max_col <- "wg"
      max_val <- numeric_data["wg"]
    } else {
      max_col <- colnames(numeric_data)[which.max(numeric_data)]
      max_val <- max(numeric_data, na.rm = TRUE)
    }

    # Store the row name, column name, and maximum value in the result matrix
    top_opsin_metagene[i,] <- c(rownames(opsinMetagenes)[i], max_col, max_val)
  } else {
    # If no numeric data in the row (or all NA values), assign NA for both column and ma
    top_opsin_metagene[i,] <- c(rownames(opsinMetagenes)[i], NA, NA)
  }
}
```

In [21]:
```
# Metagenes associated with Opsins found in the Hydra Single Cell Dataset
# Print the resulting data frame
print(top_opsin_metagene)

# Export the top_opsin_metagene data frame to a CSV file, including row names
write.csv(top_opsin_metagene, "./output/top_opsin_metagene.csv", row.names = TRUE)
```

```
    Row_Name Top_Column         Max_Value
1  g18853.t1       wg23 0.142015388708641
2  g10186.t1       wg30 0.124216173636526
3  g10185.t1       wg17 0.132939685540326
4  g30488.t1       wg63 0.154133640962899
5  g25858.t1       wg50 0.154343782056708
6  g25854.t1       <NA>              <NA>
7  g13691.t1       wg76 0.263805977917493
8   g3548.t1       <NA>              <NA>
9   g3547.t1       wg29 0.315885592889302
10  g3546.t1       wg29 0.239018118000062
11  g3536.t1       <NA>              <NA>
12  g3518.t1       <NA>              <NA>
13 g28613.t1       <NA>              <NA>
14 g28610.t1       wg22 0.177484082506587
15 g28609.t1       <NA>              <NA>
16  g1616.t1       wg23 0.259531812002445
17 g15899.t1       <NA>              <NA>
18 g15864.t1       <NA>              <NA>
19 g15863.t1       wg63 0.229893578265267
20 g17463.t2       <NA>              <NA>
21 g17463.t1       wg44   0.1526291993288
22 g25557.t1       wg16 0.252367448169969
23 g25553.t1       <NA>              <NA>
24 g25552.t1       <NA>              <NA>
25 g26211.t1       wg55 0.111458823939634
26 g29332.t1       wg21 0.250825463489907
27 g21488.t1       wg17 0.116077362706482
28         -       <NA>              <NA>
```

In [ ]:
