# Supplementary material for: Shedding Light on Patterns of Unconventional Expression of Opsin Genes in Hydra vulgaris
Source: Integr Comp Biol. 2025 Jun 23;65(3):648–60. doi: 10.1093/icb/icaf100 (PMC12464818; doi:10.1093/icb/icaf100)
Supplement: icaf100_Supplemental_Files [file icaf100_supplemental_files.zip › icb-2025-0124-File010.pdf]

Transcript ID	Augustus gene module from HGP	ATAC seq peak number	Metagene	PFAM TF annotation	Notes
t20729aep	Sc4wPfr_704.g18853.t1	64499 (DS) 64498 (US)	wg23	-	-
t20043aep	Sc4wPfr_558.1.g10186.t1	56063, 56064, 56065, 56067 (US) 56062, 56061 (DS)	wg30	-	TG1
t20044aep			wg30	-	TG1
t29150aep	Sc4wPfr_558.1.g10185.t1	56055, 56056 (US)	wg17	OTX2_XENTR, SIX6_HUMAN, DLL1_XENLA, HXA9B_TAKRU, TEF_MOUSE, EEA1_MOUSE	TG1
t25412aep	Sc4wPfr_363.3.g30488.t1	40344, 40345 (DS)	wg63	-	-
t3168aep	Sc4wPfr_338.g25858.t1	37598 (DS) 37599 (IN) 37001, 37601, 37601 (US)	wg50	-	-
t3233aep	Sc4wPfr_338.g25854.t1	37575 (US) 37577, 37578, 37579, 37580, 37581 (IN) 37582 (DS)	-	-	-
t32881aep			-	-	-
t3169aep			-	-	-
t21413aep	Sc4wPfr_303.g13691.t1	34163, 34164, 34165 (DS) 34166 (US)	wg76	RFX4_HUMAN	-
t24564aep	Sc4wPfr_200.g3548.t1	21480, 21481 (DS) 1482 (US)	-	-	TG2
t31971aep	Sc4wPfr_200.g3547.t1	21476, 21477, 21478 (US) 21479 (DS)	wg29	-	TG2
t355aep			wg29	-	TG2
t10575aep	Sc4wPfr_200.g3546.t1	21473, 21474, 21475 (US) 21476, 21477, 21478 (IN) 21479	wg29	-	TG2
t24044aep	Sc4wPfr_200.g3536.t1	21461 (US) 21462, 21463 (DS)	-	-	-
t26793aep	Sc4wPfr_200.g3518.t1	21409, 21410, 21411 (US)	-	-	-
t9221aep	Sc4wPfr_199.g28613.t1	21005 (DS)	-	-	TG3
t4885aep	Sc4wPfr_199.g28610.t1	20994 (US)	wg22	-	TG3
t15588aep			wg22	-	TG3
t24989aep	Sc4wPfr_199.g28609.t1	20992 (DS) 20993 (US)	-	-	
t27882aep			-	-	TG3
t33805aep	Sc4wPfr_172.1.g1616.t1	17064, 17065, 17066 (US) 17063 (DS)	wg23	-	-
t37969aep	Sc4wPfr_17.g15899.t1	16739, 16740, 16741, 16742, 16743 (DS)	-	-	-
t3337aep	Sc4wPfr_17.g15864.t1	16653 (US) 16654, 16655 (IN)	-	-	TG4
t36346aep	Sc4wPfr_17.g15863.t1	16646, 16647, 16648 (US)16649, 16650 (DS)	wg63	-	TG4
t33516aep	Sc4wPfr_163.g17463.t2	-	-	-	-
t33517aep	Sc4wPfr_163.g17463.t1	-	wg44	NDF1_DANRE	-
t4602aep	Sc4wPfr_161.g25557.t1	14854, 14855, 14856, 14857 (US) 14858, 14859, 14860 (DS)	wg16	-	-
t26465aep	Sc4wPfr_161.g25553.t1	14844 US 14841, 14842, 14843 (IN) 14840 (DS)	-	-	-
t26466aep			-	-	-
t29512aep	Sc4wPfr_126.g26211.t1	8408 (US) 8408 (DS)	wg55	-	-
t33568aep	Sc4wPfr_126.2.g29332.t1	8723 (US) 8724, 8725 (DS)	wg21	-	-
t14044aep			wg21	-	-

t29959aep	Sc4wPfr_1241.g21488.t1	8098, 8097, 8096, 8095, 8094 (US) 8093, 8092, 8091 (DS)	wg17	OTX2_XENTR, SIX6_HUMAN, DLL1_XENLA, HXA9B_TAKRU, TEF_MOUSE, EEA1_MOUSE	-
t17353aep	*	12391, 12392 (?)	-	-	-
t2106aep	*	-	-	-	Not on HGP
t21092aep	*	-	-	-	Not on HGP
t27688aep	*	23571, 23572 (?)	-	-	-
t36136aep	*	12443 (?)	-	-	-
t36280aep	*	38355(?) 38356 (?)	-	-	-
t4128aep	*	16720 (?) 16721, 16722, 16733 (?)	-	-	-
t32850aep	*	-	-	-	-
t31375aep			-	-	-
t20210aep	*	16658, 16659 (?) 16657 (?)	-	-	-
t16278aep	*	1497, 1498 (?)	-	-	-
