# Supplementary material for: Shedding Light on Patterns of Unconventional Expression of Opsin Genes in Hydra vulgaris
Source: Integr Comp Biol. 2025 Jun 23;65(3):648–60. doi: 10.1093/icb/icaf100 (PMC12464818; doi:10.1093/icb/icaf100)
Supplement: icaf100_Supplemental_Files [file icb-2025-0124-File007.pdf › icb-2025-0124-File007.pdf]

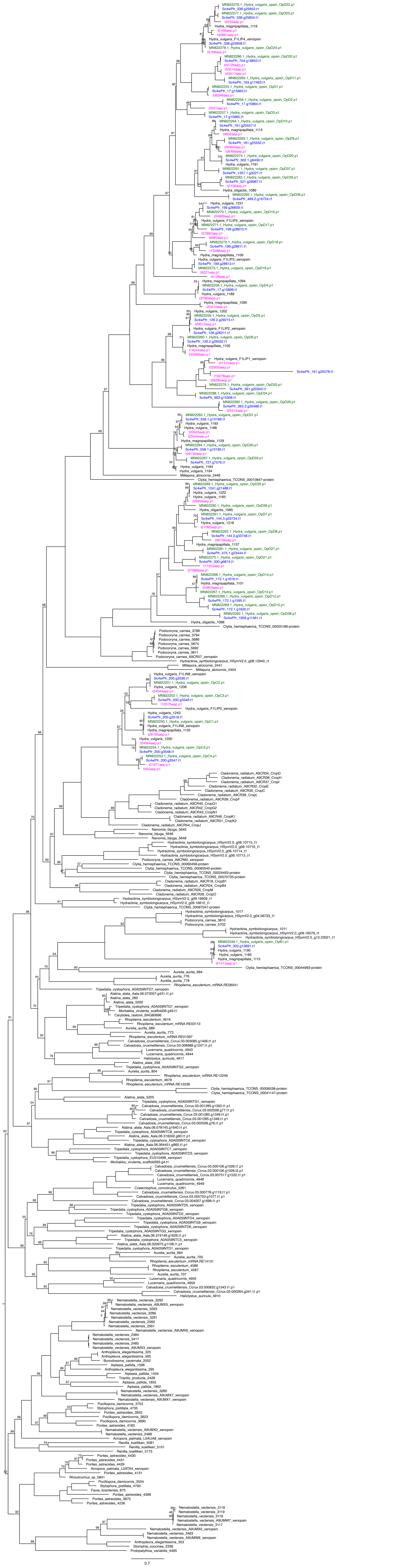

MN822276.1_Hydra_vulgaris_opsin_OpD22.p1
MN822277.1_Hydra_vulgaris_opsin_OpD23.p1
Sc4wPtr_338.g25853.t1
Sc4wPtr_338.g25854.t1
t3233aep.p1
Hydra_magnipapillata_1116
t3169aep.p1
t3281aep.p1
Hydra_vulgaris_F1LIP4_xenopsin
Sc4wPtr_338.g25858.t1
MN822278.1_Hydra_vulgaris_opsin_OpD24.p1
t3168aep.p1
MN822286.1_Hydra_vulgaris_opsin_OpD32.p1
Sc4wPtr_704.g18853.t1
t20729aep.p1
t33516aep.p1
t33517aep.p1
MN822265.1_Hydra_vulgaris_opsin_OpD11.p1
Sc4wPtr_183.g17463.t1
MN822255.1_Hydra_vulgaris_opsin_OpD1.p1
Sc4wPtr_17.g15863.t1
t36346aep.p1
MN822256.1_Hydra_vulgaris_opsin_OpD2.p1
Sc4wPtr_17.g15864.t1
t3337aep.p1
MN822257.1_Hydra_vulgaris_opsin_OpD3.p1
Sc4wPtr_17.g15865.t1
MN822264.1_Hydra_vulgaris_opsin_OpD10.p1
Sc4wPtr_161.g25557.t1
Hydra_magnipapillata_1113
t4602aep.p1
MN822263.1_Hydra_vulgaris_opsin_OpD9.p1
Sc4wPtr_161.g25552.t1
t26465aep.p1
t26466aep.p1
MN822274.1_Hydra_vulgaris_opsin_OpD20.p1
Sc4wPtr_302.1.g9439.t1
Hydra_vulgaris_1191
MN822291.1_Hydra_vulgaris_opsin_OpD37.p1
Sc4wPtr_1357.1.g3227.t1
MN822283.1_Hydra_vulgaris_opsin_OpD29.p1
Sc4wPtr_521.g26967.t1
t2106aep.p1
Hydra_oligactis_1089
MN822282.1_Hydra_vulgaris_opsin_OpD28.p1
Sc4wPtr_489.2.g16724.t1
Hydra_vulgaris_1231
Sc4wPtr_199.g28609.t1
MN822270.1_Hydra_vulgaris_opsin_OpD16.p1
t24989aep.p1
Hydra_vulgaris_F1LIP5_xenopsin
MN822271.1_Hydra_vulgaris_opsin_OpD17.p1
Sc4wPtr_199.g28610.t1
t27982aep.p1
t4885aep.p1
MN822272.1_Hydra_vulgaris_opsin_OpD18.p1
Sc4wPtr_199.g28611.t1
t1588aep.p1
Hydra_magnipapillata_1106
Hydra_vulgaris_F1LIP3_xenopsin
Sc4wPtr_199.g28613.t1
MN822273.1_Hydra_vulgaris_opsin_OpD19.p1
t9221aep.p1
t4128aep.p1
Hydra_magnipapillata_1094
MN822258.1_Hydra_vulgaris_opsin_OpD4.p1
Sc4wPtr_17.g15899.t1
Hydra_vulgaris_1189
t37969aep.p1
Hydra_magnipapillata_1090
t20210aep.p1
Hydra_vulgaris_1202
MN822259.1_Hydra_vulgaris_opsin_OpD5.p1
Sc4wPtr_126.2.g29215.t1
t29512aep.p1
Hydra_vulgaris_F1LIP2_xenopsin
Sc4wPtr_126.g26211.t1
MN822260.1_Hydra_vulgaris_opsin_OpD6.p1
Sc4wPtr_126.2.g29332.t1
Hydra_magnipapillata_1100
t14044aep.p1
t33568aep.p1
Hydra_vulgaris_F1LIP1_xenopsin
t31375aep.p1
t32830aep.p1
Sc4wPtr_161.g25578.t1
t16278aep.p1
t36280aep.p1
MN822279.1_Hydra_vulgaris_opsin_OpD25.p1
Sc4wPtr_361.g20343.t1
MN822288.1_Hydra_vulgaris_opsin_OpD34.p1
Sc4wPtr_963.g15308.t1
MN822281.1_Hydra_vulgaris_opsin_OpD26.p1
Sc4wPtr_363.3.g30488.t1
t25412aep.p1
MN822285.1_Hydra_vulgaris_opsin_OpD31.p1
Sc4wPtr_558.1.g10186.t1
Hydra_vulgaris_1193
Hydra_vulgaris_1188
t20043aep.p1
t20044aep.p1
Hydra_magnipapillata_1129
MN822284.1_Hydra_vulgaris_opsin_OpD30.p1
Sc4wPtr_558.1.g10185.t1
t29150aep.p1
MN822287.1_Hydra_vulgaris_opsin_OpD33.p1
Sc4wPtr_727.g7576.t1
Hydra_vulgaris_1164
Hydra_vulgaris_1194
Millepora_alcicornis_2446
Clytia_hemisphaerica_TCONS_00010847-protein
MN822289.1_Hydra_vulgaris_opsin_OpD35.p1
Sc4wPtr_1241.g21488.t1
Hydra_vulgaris_1222
Hydra_vulgaris_1183
t29959aep.p1
MN822290.1_Hydra_vulgaris_opsin_OpD36.p1
Hydra_oligactis_1085
MN822261.1_Hydra_vulgaris_opsin_OpD7.p1
Sc4wPtr_144.3.g33734.t1
Hydra_vulgaris_1218
t21092aep.p1
MN822262.1_Hydra_vulgaris_opsin_OpD8.p1
Sc4wPtr_144.3.g33748.t1
t36136aep.p1
Hydra_magnipapillata_1137
MN822281.1_Hydra_vulgaris_opsin_OpD27.p1
Sc4wPtr_475.1.g23444.t1
MN822275.1_Hydra_vulgaris_opsin_OpD21.p1
Sc4wPtr_330.g6874.t1
t17303aep.p1
t27688aep.p1
MN822268.1_Hydra_vulgaris_opsin_OpD14.p1
Sc4wPtr_172.1.g1616.t1
Hydra_magnipapillata_1101
t33805aep.p1
MN822267.1_Hydra_vulgaris_opsin_OpD13.p1
MN822266.1_Hydra_vulgaris_opsin_OpD12.p1
Sc4wPtr_172.1.g1595.t1
MN822269.1_Hydra_vulgaris_opsin_OpD15.p1
Sc4wPtr_172.1.g1620.t1
MN822292.1_Hydra_vulgaris_opsin_OpD38.p1
Sc4wPtr_1909.g11461.t1
Hydra_oligactis_1088
Clytia_hemisphaerica_TCONS_00025186-protein
Podocoryna_carnea_3788
Podocoryna_carnea_3794
Podocoryna_carnea_5686
Podocoryna_carnea_5674
Podocoryna_carnea_5692
Podocoryna_carnea_3811
Podocoryna_carnea_A9CR57_xenopsin
Hydractinia_symbiolongicarpus_HSymV2.0_g08.12940_t1
Millepora_alcicornis_2441
Millepora_alcicornis_2454
Hydra_vulgaris_F1LIN8_xenopsin
Sc4wPtr_200.g3536.t1
MN822251.1_Hydra_vulgaris_opsin_OpC2.p1
Hydra_vulgaris_1206
t24684aep.p1
MN822252.1_Hydra_vulgaris_opsin_OpC3.p1
Sc4wPtr_200.g3548.t1
t10575aep.p1
Hydra_vulgaris_F1LIP0_xenopsin
Hydra_vulgaris_1243
Sc4wPtr_200.g3518.t1
MN822250.1_Hydra_vulgaris_opsin_OpC1.p1
Hydra_vulgaris_F1LIN9_xenopsin
Hydra_magnipapillata_1135
t26793aep.p1
Hydra_vulgaris_1200
t24584aep.p1
MN822254.1_Hydra_vulgaris_opsin_OpC5.p1
Sc4wPtr_200.g3548.t1
MN822253.1_Hydra_vulgaris_opsin_OpC4.p1
Sc4wPtr_200.g3547.t1
t31971aep.p1
t355aep.p1
Cladonema_radiatum_A9CR34_CropD
Cladonema_radiatum_A9CR36_CropH
Cladonema_radiatum_A9CR37_CropI
Cladonema_radiatum_A9CR32_CropE
Cladonema_radiatum_A9CR30_CropC
Cladonema_radiatum_A9CR38_CropL
Cladonema_radiatum_A9CR39_CropF
Cladonema_radiatum_A9CR40_CropG1
Cladonema_radiatum_A9CR42_CropG2
Cladonema_radiatum_A9CR43_CropN1
Cladonema_radiatum_A9CR48_CropK1
Cladonema_radiatum_A9CR51_CropK2
Cladonema_radiatum_A9CR54_CropJ
Nanomia_bijuga_5645
Nanomia_bijuga_5646
Nanomia_bijuga_5648
Hydractinia_symbiolongicarpus_HSymV2.0_g06.10715_t1
Hydractinia_symbiolongicarpus_HSymV2.0_g06.10716_t1
Hydractinia_symbiolongicarpus_HSymV2.0_g06.10714_t1
Hydractinia_symbiolongicarpus_HSymV2.0_g06.10713_t1
Podocoryna_carnea_A9CR60_xenopsin
Clytia_hemisphaerica_TCONS_00068458-protein
Clytia_hemisphaerica_TCONS_00065540-protein
Clytia_hemisphaerica_TCONS_00024493-protein
Clytia_hemisphaerica_TCONS_00070735-protein
Cladonema_radiatum_A9CR18_CropB1
Cladonema_radiatum_A9CR24_CropB4
Cladonema_radiatum_A9CR26_CropM
Cladonema_radiatum_A9CR28_CropO
Hydractinia_symbiolongicarpus_HSymV2.0_g09.16609_t1
Hydractinia_symbiolongicarpus_HSymV2.0_g09.16610_t1
Clytia_hemisphaerica_TCONS_00067047-protein
Hydractinia_symbiolongicarpus_1017
Hydractinia_symbiolongicarpus_HSymV2.0_g04.06733_t1
Podocoryna_carnea_3810
Podocoryna_carnea_5702
Hydractinia_symbiolongicarpus_1011
Hydractinia_symbiolongicarpus_HSymV2.0_g09.16579_t1
Hydractinia_symbiolongicarpus_HSymV2.0_g13.23021_t1
MN822249.1_Hydra_vulgaris_opsin_OpB1.p1
Sc4wPtr_303.g13691.t1
Hydra_vulgaris_1190
Hydra_vulgaris_1185
Hydra_magnipapillata_1115
t21413aep.p1
Clytia_hemisphaerica_TCONS_00044993-protein
Aurelia_aurita_684
Aurelia_aurita_776
Aurelia_aurita_778
Rhopilema_esculentum_mRNA.RE06541
Tripedalia_cystophora_A0A059NTD7_xenopsin
Alatina_alata_Aala.06.07307.g431.t1.p1
Alatina_alata_260
Alatina_alata_5200
Tripedalia_cystophora_A0A059NTG7_xenopsin
Morbakka_virulenta_scaffold36.g49.t1
Carybdea_rastonii_BAG80696
Rhopilema_esculentum_4616
Rhopilema_esculentum_mRNA.RE03113
Aurelia_aurita_689
Aurelia_aurita_773
Rhopilema_esculentum_mRNA.RE01397
Calvadosia_cruxmelitensis_Corux.03.003085.g1406.t1.p1
Calvadosia_cruxmelitensis_Corux.03.008988.g1247.t1.p1
Lucernaria_quadricornis_4943
Lucernaria_quadricornis_4944
Haliclystus_auricula_4917
Alatina_alata_256
Tripedalia_cystophora_A0A059NTG2_xenopsin
Aurelia_aurita_804
Rhopilema_esculentum_mRNA.RE12249
Rhopilema_esculentum_4679
Rhopilema_esculentum_mRNA.RE12236
Clytia_hemisphaerica_TCONS_00006538-protein
Clytia_hemisphaerica_TCONS_00041147-protein
Alatina_alata_5205
Tripedalia_cystophora_A0A059NTG1_xenopsin
Calvadosia_cruxmelitensis_Corux.03.001285.g1350.t1.p1
Calvadosia_cruxmelitensis_Corux.03.002508.g77.t1.p1
Calvadosia_cruxmelitensis_Corux.03.001285.g1349.t1.p1
Calvadosia_cruxmelitensis_Corux.03.001285.g1348.t1.p1
Calvadosia_cruxmelitensis_Corux.03.002508.g78.t1.p1
Alatina_alata_Aala.06.078145.g1640.t1.p1
Tripedalia_cystophora_A0A059NTC8_xenopsin
Alatina_alata_Aala.06.316002.g60.t1.p1
Tripedalia_cystophora_A0A059NTC6_xenopsin
Alatina_alata_Aala.06.064451.g960.t1.p1
Tripedalia_cystophora_A0A059NTC7_xenopsin
Tripedalia_cystophora_A0A059NTD3_xenopsin
Tripedalia_cystophora_EU310498_xenopsin
Morbakka_virulenta_scaffold393.g4.t1
Calvadosia_cruxmelitensis_Corux.03.000106.g1028.t1.p1
Calvadosia_cruxmelitensis_Corux.03.000106.g1028.t2.p1
Calvadosia_cruxmelitensis_Corux.03.007517.g1532.t1.p1
Lucernaria_quadricornis_4948
Lucernaria_quadricornis_4949
Craterolophus_convolvulus_5261
Calvadosia_cruxmelitensis_Corux.03.000778.g1119.t1.p1
Calvadosia_cruxmelitensis_Corux.03.005753.g1077.t1.p1
Calvadosia_cruxmelitensis_Corux.03.004007.g1696.t1.p1
Tripedalia_cystophora_A0A059NTD5_xenopsin
Tripedalia_cystophora_A0A059NTG8_xenopsin
Tripedalia_cystophora_A0A059NTD2_xenopsin
Tripedalia_cystophora_A0A059NTD4_xenopsin
Tripedalia_cystophora_A0A059NTG9_xenopsin
Tripedalia_cystophora_A0A059NTD6_xenopsin
Tripedalia_cystophora_A0A059NTG3_xenopsin
Alatina_alata_Aala.06.319149.g1626.t1.p1
Tripedalia_cystophora_A0A059NTC5_xenopsin
Alatina_alata_Aala.06.020975.g1108.t1.p1
Tripedalia_cystophora_A0A059NTD1_xenopsin
Aurelia_aurita_664
Aurelia_aurita_705
Rhopilema_esculentum_mRNA.RE14131
Rhopilema_esculentum_4586
Rhopilema_esculentum_4587
Aurelia_aurita_707
Lucernaria_quadricornis_4955
Lucernaria_quadricornis_4959
Calvadosia_cruxmelitensis_Corux.03.000832.g1343.t1.p1
Calvadosia_cruxmelitensis_Corux.03.000264.g341.t1.p1
Haliclystus_auricula_4910
Nematostella_vectensis_3292
Nematostella_vectensis_A9UMX5_xenopsin
Nematostella_vectensis_3293
Nematostella_vectensis_3289
Nematostella_vectensis_3291
Nematostella_vectensis_2560
Nematostella_vectensis_2561
Nematostella_vectensis_A9UMX6_xenopsin
Nematostella_vectensis_2484
Nematostella_vectensis_3417
Nematostella_vectensis_2485
Nematostella_vectensis_A9UMX3_xenopsin
Anthopleura_elegantissima_325
Anthopleura_elegantissima_565
Bunodosoma_cavernata_2052
Aiptasia_pallida_1596
Anthopleura_elegantissima_295
Aiptasia_pallida_1434
Triactis_producta_2428
Aiptasia_pallida_1855
Aiptasia_pallida_1862
Nematostella_vectensis_3260
Nematostella_vectensis_A9UMX7_xenopsin
Nematostella_vectensis_A9UMX1_xenopsin
Pocillopora_damicornis_3753
Stylophora_pistillata_4735
Porites_astreoides_3952
Pocillopora_damicornis_3623
Pocillopora_damicornis_3690
Porites_astreoides_4183
Nematostella_vectensis_A9UMX2_xenopsin
Nematostella_vectensis_2488
Acropora_palmata_L0AUA8_xenopsin
Renilla_koellikeri_5081
Renilla_koellikeri_5101
Renilla_koellikeri_5175
Porites_astreoides_4430
Porites_astreoides_4431
Porites_astreoides_4429
Acropora_palmata_L0ATA4_xenopsin
Porites_astreoides_4131
Rhizotrochus_sp_5841
Pocillopora_damicornis_3554
Stylophora_pistillata_4700
Favia_lizardensis_875
Porites_astreoides_4399
Porites_astreoides_3875
Porites_astreoides_4236
Nematostella_vectensis_3116
Nematostella_vectensis_3119
Nematostella_vectensis_3118
Nematostella_vectensis_A9UMW7_xenopsin
Nematostella_vectensis_3117
Nematostella_vectensis_A9UMX0_xenopsin
Nematostella_vectensis_3463
Nematostella_vectensis_A9UMW6_xenopsin
Anthopleura_elegantissima_353
Stomphia_coccinea_2336
Protopalythoa_variabilis_4495
0.7
