# Supplementary material for: Shedding Light on Patterns of Unconventional Expression of Opsin Genes in Hydra vulgaris
Source: Integr Comp Biol. 2025 Jun 23;65(3):648–60. doi: 10.1093/icb/icaf100 (PMC12464818; doi:10.1093/icb/icaf100)

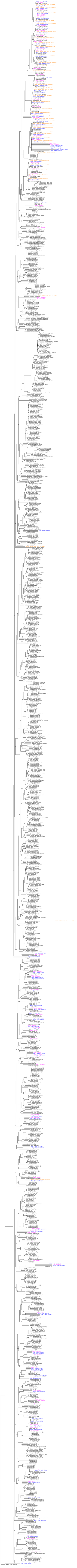

Supplement: icaf100_Supplemental_Files [file icb-2025-0124-File008.pdf › icb-2025-0124-File008.pdf]
